# The Interplay between Ferroptosis and Neuroinflammation in Central Neurological Disorders

**DOI:** 10.3390/antiox13040395

**Published:** 2024-03-26

**Authors:** Yejia Xu, Bowen Jia, Jing Li, Qianqian Li, Chengliang Luo

**Affiliations:** 1 Department of Forensic Medicine, School of Basic Medicine and Biological Sciences, Soochow University, Suzhou 215123, China; 2 Hebei Key Laboratory of Forensic Medicine, College of Forensic Medicine, Hebei Medical University, Shijiazhuang 050017, China; 3 NHC Key Laboratory of Drug Addiction Medicine, Department of Forensic Medicine, School of Forensic Medicine, Kunming Medical University, Kunming 650500, China; 4 School of Forensic Medicine, Wannan Medical College, Wuhu 241002, China

**Keywords:** ferroptosis, neuroinflammation, central neurological disorders, blood–brain barrier

## Abstract

Central neurological disorders are significant contributors to morbidity, mortality, and long-term disability globally in modern society. These encompass neurodegenerative diseases, ischemic brain diseases, traumatic brain injury, epilepsy, depression, and more. The involved pathogenesis is notably intricate and diverse. Ferroptosis and neuroinflammation play pivotal roles in elucidating the causes of cognitive impairment stemming from these diseases. Given the concurrent occurrence of ferroptosis and neuroinflammation due to metabolic shifts such as iron and ROS, as well as their critical roles in central nervous disorders, the investigation into the co-regulatory mechanism of ferroptosis and neuroinflammation has emerged as a prominent area of research. This paper delves into the mechanisms of ferroptosis and neuroinflammation in central nervous disorders, along with their interrelationship. It specifically emphasizes the core molecules within the shared pathways governing ferroptosis and neuroinflammation, including SIRT1, Nrf2, NF-κB, Cox-2, iNOS/NO·, and how different immune cells and structures contribute to cognitive dysfunction through these mechanisms. Researchers’ findings suggest that ferroptosis and neuroinflammation mutually promote each other and may represent key factors in the progression of central neurological disorders. A deeper comprehension of the common pathway between cellular ferroptosis and neuroinflammation holds promise for improving symptoms and prognosis related to central neurological disorders.

## 1. Introduction

Nerve cell death stands as a significant contributor to neurological disorders, diseases, and injuries. The unexpected or programmed demise of neurons and glial cells across various regions of the nervous system disrupts sensory, motor, cognitive, learning, and memory functions [1,2,3]. Ferroptosis, a form of regulated cell death triggered by iron overload, involves distinct inducible factors and metabolites [4]. Since its inception in 2012, research has revealed that cellular ferroptosis can occur ubiquitously throughout the human body, including within the nervous system. Imbalances in iron metabolism prompt nerve cells to generate high levels of reactive oxygen species via the Fenton reaction, leading to lipid peroxidation and impairment of cellular REDOX functions [5]. Furthermore, ferroptosis-induced mitochondrial atrophy and functional decline impede the energy supply to neurons, disrupting electrical and chemical signal transmission [6]. The ferroptosis of nerve cells within the central nervous system undermines cellular physiological functions, with cognitive impairment emerging as a hallmark mechanism in central nervous system diseases and injuries.

Neuroinflammation represents a unique inflammatory response within the human body, serving as the central nervous system’s defense mechanism against diverse stimuli [7]. This process involves the activation of microglia and astrocytes, the primary immune-active cells, which release various cytokines and chemokines [8,9]. In the context of central nervous system diseases and injuries, neuroinflammation is a common occurrence, leading to the release of numerous effector proteins that disrupt neuronal excitability, thereby exacerbating nerve function impairment [10].

The coexistence of neuroinflammation and cell ferroptosis in neuropathy has sparked significant interest in recent years within the study of central nervous system diseases. Common inflammatory factors released during neuroinflammation exhibit close associations with proteins that govern cellular iron metabolism and lipid metabolism [11]. In light of this, researchers are delving into the potential links and shared pathways between neuroinflammation and ferroptosis by uncovering the molecular mechanisms that underlie diverse structural alterations within cells following central nervous system diseases and injuries.

## 2. Ferroptosis and Central Neurological Disorders

### 2.1. Mechanism and Pathway of Ferroptosis

Ferroptosis is a newly discovered type of programmed cell death, first identified in 2012 by Drs. Brent R. Stockwell, Scott Dixon, and their research team [4]. It is considered an independent mechanism of cell death because cells undergoing ferroptosis exhibit distinct morphological, compositional, and metabolic characteristics compared to common forms of cell death, such as apoptosis and necrosis. Morphologically, cells undergoing ferroptosis do not display fragmented or shrunken nuclei but instead exhibit atrophied mitochondria and a reduced number of cristae [12]. In the cytoplasmic component, the levels of free iron and reactive oxygen species are elevated in these cells [13].

The cellular metabolism involved in ferroptosis encompasses various facets, including abnormalities in iron homeostasis, lipid metabolism, and glutathione-related metabolic changes [4]. These alterations in metabolism often serve as critical targets for the regulation of ferroptosis. Pertaining to iron metabolism, cells undergoing ferroptosis exhibit modifications in the processes of iron uptake, storage, utilization, and efflux, culminating in cellular iron overload [14,15]. Researchers have addressed the issue of elevated cellular iron levels by regulating proteins such as transferrin receptor, ferritin, ferroportin1, and employing iron chelators like deferriamine [14,16,17]. In the realm of cellular lipid metabolism, the peroxidation of polyunsaturated fatty acids into PUFA-PLs represents a pivotal pathway in ferroptosis [4]. Building upon this foundation, strategies such as inhibiting PUFAacyl-CoA derivatives (PUFA-CoAs) and ACSL4, a key enzyme generated by lipoxygenase, or promoting monounsaturated fatty acids (MUFAs) over PUFAs have been explored as means to impede ferroptosis [18,19,20]. Concerning intracellular REDOX metabolism, diverse antioxidant pathways have been identified as capable of combating the progression of cellular ferroptosis. Apart from the well-known GPX4 classical pathway, recent discoveries have unveiled the potential of pathways like FSP1/CoQ10, DHODH, and GCH1/BH4 in inhibiting intracellular peroxidation by reducing CoQ10 at the plasma membrane, converting CoQ to panthenol, and sequestering free radicals, respectively [21,22,23]. Ongoing research continues to enhance our understanding of the mechanisms and regulation of ferroptosis, while investigations into its interplay with other cell death mechanisms and additional metal elements such as copper and zinc offer novel avenues for exploration [24].

### 2.2. Ferroptosis in Central Neurological Disorders

The typical progression of ferroptosis has been discovered in various systemic diseases. Over the past decade, researchers have discovered that ferroptosis plays a crucial role in many central nervous system diseases and injuries, including neurodegenerative diseases, stroke, epilepsy, brain injury, and depression. In the early stages of research, significant iron content and elevated transferrin levels were observed in the local blood vessels and brain parenchyma of ischemic stroke [25]. With the recognition of ferroptosis, studies have found that ischemic stroke regulation of ACSL4 can change the pathologic severity through the pathway of ferroptosis [26]. Alzheimer’s disease is characterized by the deposition of β-amyloid protein (Aβ) plaques and neurofibrillary tangles (NFTs) in the brain, which are key pathological features. There is evidence suggesting that ferroptosis in the Alzheimer’s disease (AD) brain exhibits a unique manifestation: the abnormal elevation of xCT exacerbates neuroexcitatory toxicity and amyloid-beta (Aβ) accumulation, ultimately impairing the neural function in individuals with AD [27]. Parkinson’s disease is marked by symptoms such as resting tremors resulting from the degeneration of neurons in the substantia nigra of the midbrain. Research indicates that dopamine in the brain could potentially have a positive regulatory effect on GPX4. Therefore, the depletion of dopamine in the brain of individuals with Parkinson’s disease (PD) may lead to ferroptosis due to the loss of GPX4 [28]. Furthermore, in epileptic brains, astrocytes modulate xCT/GSH/GPX4 via chemokines, leading to neuronal ferroptosis, possibly associated with synchronous brain firing processes [29]. Traumatic brain injury (TBI) is a major cause of death and disability in developed countries. The expression of TFR, FPN, and GPX4 exhibited varying degrees of increase at 6 h, 1 day, 3 days, 7 days, and 3 weeks in the brain tissue of the injured area. Additionally, it has been observed that ferroptosis-specific inhibitors can effectively inhibit the extent of secondary damage following TBI [30].

The discovery of the various diseases mentioned above highlights that dysregulation of iron metabolism, lipid metabolism, and abnormalities in GPX4-related pathways in the affected areas have become common features of brain neurological diseases. Ferroptosis plays a significant role in the pathogenesis of central nervous system diseases, and interventions targeting ferroptosis have become crucial strategies for combating these diseases. By focusing on modulating ferroptosis and related pathways, researchers aim to develop novel therapeutic approaches to address the underlying mechanisms of these neurological disorders and potentially improve patient outcomes.

### 2.3. Cognitive Dysfunction Caused by Ferroptosis

The cognitive function of mammals relies on the integrity of structures such as the cerebral cortex, hippocampus, and basal ganglia. Cognitive dysfunction is a primary symptom of central nervous system diseases and serves as a key indicator for assessing therapeutic effects and evaluating prognosis in numerous conditions. Neuronal damage in various brain regions stands as the fundamental cause of cognitive impairment. Prior to the definition of ferroptosis, numerous studies had revealed a distinct spatial and temporal correlation between iron overload and cognitive dysfunction in the brain [31]. In addition to discovering in mouse models that a high-iron diet during the neonatal period led to cognitive impairment in adulthood, researchers also observed that iron overload in various regions of the human brain worsened cognitive dysfunction in neurological diseases [32]. The inhibition of elevated iron levels in certain neurodegenerative disease models through iron chelators has proven effective in reducing cognitive impairment [31].

In recent years, ferroptosis has been identified as prevalent in a range of central nervous system diseases, causing damage to nerve cells and ultimately leading to neuronal death and cognitive dysfunction. Mei and Liu’s research team illustrated that Nrf2-dependent cortical neuronal ferroptosis induces severe cognitive impairment in mice, as evidenced by Morris water maze tests, Rasin scores, and electroencephalograms of epileptic mice [33]. Studies have indicated a positive correlation between the extent of cognitive impairment resulting from traumatic brain injury, sepsis-associated epilepsy, and epilepsy and the degree of ferroptosis in hippocampal neurons [34,35,36]. Parkinson’s disease stems from the ferroptosis of neurons in the substantia nigra compacta (SNpc), leading to motor dysfunction like static tremors, as well as subcortical cognitive deficits such as executive dysfunction and attention deficit [37]. The inhibition of ferroptosis to ameliorate cognitive impairment in central nervous system diseases represents a current focal point in the treatment of diverse conditions.

## 3. Neuroinflammation in Central Neurological Disorders

Neuroinflammation is essentially an immune response triggered by physiological abnormalities or pathological conditions of the central nervous system. It is observed in nearly all cases of central nervous dysfunction, including aging, infectious brain diseases, autoimmune encephalopathy, neurodegenerative diseases, ischemic stroke, and brain injury [38,39,40,41,42,43]. The primary effector cell phenotypes of neuroinflammation are two centrally located innate immune cells: microglia and astrocytes [44]. Furthermore, when the structure and function of the blood–brain barrier, the centerpiece of immune privilege in defending the brain, is compromised, peripheral immune cells (such as neutrophils, monocytes/macrophages, lymphocytes, etc.) invade the brain and contribute to the amplification of neuroinflammation [45].

The initiation of neuroinflammation is typically accompanied by the injury of endogenous nerve cells or the invasion of exogenous infectious agents and toxins. Similar to immune processes in other systems, damaged or dead cells and invading infectious agents in the brain release damage-associated molecular patterns (DAMPs) and pathogen-associated molecular patterns (PAMPs), respectively [46,47]. Microglia and astrocytes in the brain are activated by receptors to become reactive microglia and reactive astrocytes [48,49]. These activated innate immune cells secrete a variety of cytokines in response to specific phenotypic changes, including interleukins, interferons, tumor necrosis factors, chemokines, and reactive oxygen species, which contribute to the amplification of neuroinflammation [50]. When pro-inflammatory cytokines take precedence, they trigger cell death, affecting neurons, glial cells, and endothelial cells. Destruction of the endothelial cells of the blood–brain barrier leads to the invasion of peripheral immune cells into the brain at different stages of the disease [51]. These infiltrating peripheral cells further disrupt the integrity of the brain structure, harm neurons, and worsen the organic brain damage caused by neuroinflammation [52].

Neuroinflammation causes damage to neurons in the affected region, including axonal degeneration, abnormal energy metabolism, destruction of synaptic structures, and even neuronal death [44]. Additionally, under pathological conditions, astrocytes and microglia lose their original physiological functions, such as neurotrophic function and maintenance of synaptic plasticity [53]. The reduced level of brain-derived neurotrophic factor (BDNF) in the brain inhibits the repair of neural synapses [54]. These consequences lead to the disruption of nerve impulse transmission, ultimately resulting in impaired cognitive function and a series of brain functional abnormalities.

## 4. The Link between Neuroinflammation and Ferroptosis

Recent studies focusing on the brain and spinal cord have illuminated the interconnected nature of cell ferroptosis and neuroinflammation in the realm of nerve injury and degeneration. Intracellular oxidative stress stemming from metabolic irregularities during ferroptosis, coupled with disruptions in mitochondrial structure and function, can exacerbate neuroinflammation [55,56,57]. The altered phenotypes of immune cells during inflammation and the release of pro-inflammatory factors can trigger ferroptosis in both immune cells themselves and neighboring neurons [58]. Thus, exploring the shared molecular communication processes between cellular ferroptosis and neuroinflammation offers valuable insights into the pathogenesis of diverse central nervous system disorders, identifies potential therapeutic targets, and enhances the management of cognitive, sensory, and motor symptoms associated with these conditions. This chapter delves into the converging pathways of ferroptosis and neuroinflammation across various central nervous system diseases, examining cell phenotypes, specific structures, and microscopic molecules to unveil commonalities and potential treatment avenues.

### 4.1. Molecular Mechanisms and Signaling Pathways

#### 4.1.1. SIRT1

Silent information regulator 1 (SIRT1) is a class III histone deacetylase that is widely expressed in mammals. Recent research has convincingly demonstrated its close association with cellular energy metabolism, inflammation, and various programmed cell death pathways. The biological activity of SIRT1 requires the presence of NAD+ and responds to changes in energy status by regulating the intracellular NAD+/NADH ratio [59,60]. Due to its exceptional performance in the body’s defense mechanisms and death modes such as oxidative stress, inflammation, apoptosis, and ferroptosis, the role of SIRT1 in the pathological process of central nervous system disease and injury is worth inferring.

The expression of SIRT1 is intricately linked to AMP-activated protein kinase (AMPK). Activation of AMPK can enhance SIRT1 activity in a NAD+-dependent manner, thereby inhibiting downstream inflammatory pathways and ferroptosis pathways [61,62]. Moreover, SIRT1 has the ability to elevate AMPK levels through acetylation modification [63]. Consequently, the reciprocal activation and regulation of AMPK and SIRT1 may establish a positive feedback loop within cells, serving as a mechanism for the body to counteract physiological dysfunction. Additionally, both AMPK and SIRT1 possess the capability to modulate gene expression by co-regulating the activity of transcription factors, including the mitochondrial functional protein PGC-1α [64,65]. PGC-1α is likely a pivotal target of SIRT1 due to its transcriptional regulation, with the associated transcription factors encompassing PPARα, PPARγ, FoxO1, Nrf1, Nrf2, etc. [66,67,68,69].

**Figure 1:**
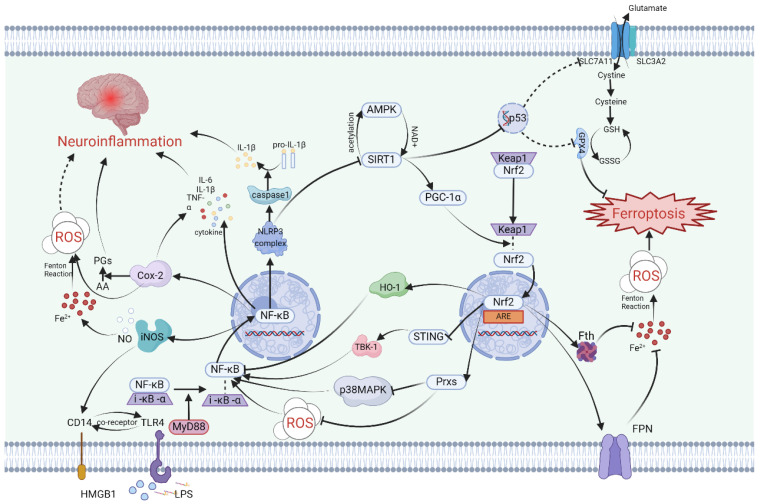
A schematic summary of the link between inflammation and ferroptosis in nerve cells. SIRT1, Nrf2, and NF-κB intricately regulate one another, thereby influencing the magnitude of neuroinflammation and ferroptosis. SIRT1 and AMPK mutually boost each other’s expression, hastening the dissociation of Nrf2 and Keap1 through the upregulation of PGC-1α. Subsequently, Nrf2 translocates into organelles and binds to ARE, thereby orchestrating the transcriptional regulation of various genes. Nrf2 can impede NF-κB activity via multiple pathways including the Nrf2/HO-1 pathway, STING/TBK-1 pathway, and Prxs/P38MAPK pathway. NF-κB, a pivotal transcription factor in inflammation regulation, not only triggers the NLRP3 complex for proinflammatory cytokine release but also suppresses SIRT1 expression. Furthermore, SIRT1 inhibits p53 gene expression, indirectly thwarting cellular ferroptosis through the GPX4 pathway. By modulating Prxs and Fth/FPN, Nrf2 governs cellular oxidative stress and labile iron pools, thereby suppressing ferroptosis. NF-κB also stimulates Cox-2 and iNOS production, influencing neuroinflammation and ferroptosis occurrence by impacting iron/ROS levels and inflammatory cytokines. Abbreviations: AA, arachidonic acid; AMPK, adenosine 5’-monophosphate (AMP)-activated protein kinase; ARE, antioxidant responseelement; FPN, ferroportin; Fth, ferritin heavy; GPX4, glutathione peroxidase 4; GSH, glutathione; GSSG, oxidized glutathione; HMGB1, high mobility group protein B1; HO-1, heme oxygenase 1; LPS, lipopolysaccharide; MAPK, mitogen-activated protein kinase; MyD88, Myeloid Differentiation Factor 88; NF-κB, nuclear factor-kappaB; Nrf2, nuclear factor erythroid-2-related factor 2; PGs, prostaglandins; PGC-1α, PPAR-γ Coactivator 1 alpha; ROS, reactive oxygen species; SIRT1, silent information regulator 1; STING, stimulator of interferon genes; TBK-1, TANK-binding kinase 1; TLR4, Toll-like receptor 4. Solid arrows indicate facilitation of expression or relocation. Dashed arrows suggest potential facilitation of expression but with unclear mechanisms. Inhibition arrows signify suppression of expression. Dashed inhibition arrows indicate potential suppression of expression but with unclear mechanisms. This figure was created with BioRender.com.

Firstly, the mechanism by which SIRT1 repairs the damaged nervous system involves the participation of Nrf2. Specifically, studies have indicated that following traumatic brain injury, SIRT1 positively regulates Nrf2 concentration, inducing the expression of Prxs (an ROS management system) and ultimately inhibiting p38 MAPK (Figure 1). This modulation not only reduces acute neurotrauma after brain injury but also improves cognitive function and long-term prognosis [70,71,72]. P38 is a serine/threonine protein kinase that plays a crucial role in cell apoptosis/inflammatory response and the nervous system [73,74]. Additionally, it has been demonstrated that two SIRT1 agonists, astaxanthin and berberine, promote cognitive and memory recovery in mice through the SIRT1/p38 MAPK pathway [70,75]. Furthermore, the SIRT1/Nrf2 pathway mitigates ferroptosis after brain injury by activating GPX4, a key factor in ferroptosis [76,77]. Upregulation of SIRT1 can reverse the decrease in GPX4 content in the hippocampus after ischemic hypoxic injury, which is an important treatment target for HIBI [76]. Surprisingly, in some inflammatory model diseases, intervention-induced activation of the SIRT1/Nrf2/GPX4 pathway has been shown to alleviate inflammatory factors, suggesting a possible role of this pathway in neuroinflammation [78,79,80].

Secondly, SIRT1 is also involved in a cascade relationship with the important human tumor suppressor gene p53, impacting SLC7A11, which increases intracellular and extracellular glutamate and cystine exchange, indirectly influencing GPX4’s inhibition of ferroptosis (Figure 1). Knocking out SIRT1 and using ferrostatin-1, a specific inhibitor of ferroptosis, have demonstrated similar inhibitory effects in the LPS-induced ferroptosis model [81]. Studies on the acetylation levels of SIRT1 and P53 K382 after Baicalein treatment have shown opposite trends [82], indicating that upregulation of SIRT1 may enhance the expression of SLC7A11 by inhibiting the p53 gene. In brain injury resulting from cerebral ischemia-reperfusion, a type of RNA-binding protein called pumilio 2 has been found to decrease the expression of SLC7A11 by inhibiting SIRT1, leading to neuroinflammation and ferroptosis in I/R [83]. It can be inferred that the SIRT1/p53/SLC7A11/GPX4 pathway may play a key role in inducing a series of ferroptosis and neuroinflammatory effects in the pathological processes of certain central nervous system diseases (Figure 1). Notably, due to the distinct role of p53 in the cell cycle, the involvement of p53 in ferroptosis directly inhibited by GPX4 differs from ferroptosis directly inhibited by SLC7A11 [84,85].

Furthermore, SIRT1 has been found to inhibit ferroptosis in nerve cells through a non-GPX4-dependent ferroptosis protection pathway. In experiments with HT22 cells in vivo and in vitro in Alzheimer’s mice, Jiatong Zhang and Wei Li’s team demonstrated that the upregulation of SIRT1 in AD inhibits ferroptosis via the FSP1 axis [86]. Similar results were observed in a study on subarachnoid hemorrhage [87]. Moreover, a test of methyl naphthoquinone-4 (MK-4) suggests that SIRT1 may also protect neurons from ferroptosis in subarachnoid hemorrhage by increasing DHODH levels [88].

Finally, SIRT1 plays a crucial role in attenuating various neuroinflammatory cascades by downregulating nuclear factor-κB (NF-κB), a central molecule in inflammation (Figure 1). The regulatory function of SIRT1 in inflammation can involve HMGB1 and Toll-like receptors (TLR4) [89]. In cases of brain injury, SIRT1 is involved in reducing NF-κB levels through the HMGB1/TLR4 pathway [90] and inhibiting the expression of NLRP3 inflammasomes [61]. The downstream inflammatory pathway associated with NF-κB activation can result in the upregulation of various pro-inflammatory factors and cell phenotype transformations, such as microglia and astrocyte activation [59], M1 polarization of microglia [91], and an increase in TNF-α, IL-1β, IL-6, and other inflammatory mediators [59,91]. Numerous biomolecules activated by SIRT1 work to reduce NF-κB expression, thereby helping to alleviate neuroinflammation in the brain [59,60,89,92].

It is evident that the activation of silent information regulator 1 can trigger both ferroptosis and inflammation in cells, usually through upstream regulatory means. As such, upregulation of SIRT1 has emerged as a potential therapeutic target for the treatment of neurological disorders and cognitive impairment in the brain. Resveratrol, a classic SIRT1 activator, has been widely used to reduce inflammation and cell death in diseases [89,93,94,95]. However, there is still much to learn about the mechanism by which SIRT1 regulates ferroptosis in nerve cells and inhibits the release of pro-inflammatory factors. For instance, the specific intermolecular processes by which SIRT1 regulates FSP1 and DHODH are not yet fully understood and disease models targeting these two atypical ferroptosis defense mechanisms are limited. Furthermore, the SIRT1/p53 pathway has different direct effects on GPX4 and SLC7A11, and the feedback relationship between p53, GPX4, and SLC7A11 requires further investigation.

#### 4.1.2. Nrf2

The nuclear factor erythroid 2-related factor 2 (Nrf2) is a DNA-binding protein that has its origins in the study of red blood cell production. While initial research on Nrf2 focused on its involvement in regulating globin gene expression, subsequent studies have highlighted its crucial role in combating oxidative stress [96]. Nrf2 accomplishes this by activating a variety of genes through binding to antioxidant response elements (AREs), particularly promoting the expression of heme oxygenase-1 (HO-1) (Figure 1). This activation of downstream pathways leads to an enhanced antioxidant response in different types of cells. 

The regulation of Nrf2 activity encompasses keap1-dependent and keap1-independent pathways, with the keap1-Cul3-Rbx1 pathway playing a pivotal role. Upon exposure to oxidative stress, the dissociation of keap1 from Nrf2 results in enhanced stability of Nrf2 and its subsequent translocation into the nucleus to bind ARE [96]. In the context of cerebral ischemia-reperfusion injury, the NLRP3 inflammasome can trigger downstream antioxidant responses and influence ferroptosis by upregulating Nrf2 induced by keap1 as a defensive reflex (Figure 1).

There are two primary forms of keap1-independent regulation. The first is the PI3K/Akt-dependent pathway, which is activated in response to oxidative stress [97]. In mouse HT22 cells, blocking the PI3K/Akt pathway hinders the antioxidant effect induced by Nrf2, ultimately exacerbating neuroinflammation [98]. In cases of traumatic brain injury (TBI), activation of the TrkB receptor upregulates Nrf2 through the PI3K/Akt pathway, thereby reducing ferroptosis and TBI-related neuroinflammation. This process also partially restores neurocognitive impairment. Consequently, the TrkB activator NAS has emerged as an effective therapeutic target [99]. The second type involves the Hrd1-dependent pathway, where Hrd1 binds to Nrf2, leading to its ubiquitination and subsequent degradation. Consequently, this inhibits the cellular protective response [100].

Nrf2, upon translocating into the nucleus, plays a crucial role in the regulation of more than 250 genes by binding to their specific sites. These genes are involved in various cellular processes such as oxidative stress, ferroptosis, autophagy, and inflammation [101]. In the context of ferroptosis, Nrf2 not only modulates intracellular iron, lipid, and redox metabolism but also exerts influence on key ferroptosis inhibition pathways like GPX4 and FSP1 through intricate molecular interactions [96,102]. Reactive oxygen species (ROS) generated during cellular stress trigger the activation of Nrf2 via the keap1-dependent pathway [103]. And then, Nrf2 reduces intracellular iron by increasing the expression of Fth and FPN, utilizing iron storage and iron effluence, respectively, and inhibits glia-driven neuronal ferroptosis by positively regulating GPX4 and FSP1 pathways [104,105]. Netrin-1, a binding protein associated with nerve regeneration, regulates ferroptosis after brain injury by influencing Nrf2 through neurite inducible factor receptor UNC5B, providing a therapeutic direction for subsequent nerve recovery [106]. The peroxisome proliferator-activated receptor PPARγ pathway, which is associated with lipid metabolism, complements Nrf2 and significantly reduces ferroptosis and cognitive impairment in pathological models of cerebral hemorrhage and cerebral ischemia [98,107,108]. 

In addition to its role in cell death and oxidative stress, recent research has highlighted Nrf2’s involvement in neuroinflammation. Studies on cognitive function in offspring rats subjected to maternal sleep deprivation have demonstrated that the ferroptosis inhibitor liproxstatin-1 can reduce the release of inflammatory factors and the activation of microglia via the Nrf2/HO-1 axis [109]. Similarly, in patients with neuroinflammation due to interstitial cystitis and in models of RSL3-induced ferroptosis, Nrf2 has been observed to be associated with pro-inflammatory cytokines and microglia [110,111]. In cases of traumatic brain injury, Nrf2 promotes mitochondrial function recovery, enhances antioxidant capacity, and inhibits neuroinflammation [112]. The mechanism by which Nrf2 regulates neuroinflammation is mainly dependent on its inhibition of downstream inflammatory pathways by influencing the activation of NF-κB (Figure 1). Nrf2 inhibits NF-κB through various means, including the induction of a shift in microglia from a pro-inflammatory M1 phenotype to an anti-inflammatory M2 phenotype via the Nrf2/STING/NF-κB pathway [113,114]. The stimulator of interferon genes (STING), which serves as an immune sensor of endogenous and exogenous DNA, may act as an intermediary in the NF-κB induction by Nrf2 (Figure 1). While the relationship between Nrf2 and STING has been confirmed by the STRING database, the cascading relationship between them remains to be elucidated [115]. The NRF2-STING axis plays a key role in regulating oxidative stress, inflammation, and necrosis [116]. Activated STING can recruit TANK-binding kinase 1 (TBK1) to accelerate the phosphorylation of NF-κB [117]. In addition, heme oxygenase HO-1, the main regulatory enzyme of Nrf2, is believed to reduce NF-κB activity [118]. Annexin A5 facilitates phenotype transformation of microglia via the Nrf2 pathway to NF-κB, counteracting the inflammatory response, ferroptosis, and oxidative stress, thereby improving neurocognitive dysfunction [119]. The effect of Nrf2 on neuroinflammation heavily relies on NF-κB, but the mechanisms by which Nrf2 regulates NF-κB are varied. The association between these two molecules remains incomplete, and the Nrf2/STING/TBK1/NF-κB pathway in the brain requires more detailed detection of protein expression and pathological experiments for verification. In particular, the indirect relationship between Nrf2 and STING needs further elaboration.

In summary, Nrf2, a key player in ferroptosis and oxidative stress, is significantly involved in the connection between neuroinflammation and ferroptosis in the central nervous system. However, our understanding of the molecular mechanisms underlying neuroinflammation remains partial and incomplete. The downstream pathway activated by Nrf2 within the complete neuroinflammatory signaling network should be viewed as three-dimensional rather than linear, requiring further in-depth exploration and discussion.

#### 4.1.3. NF-κB

Nuclear factor-κB (NF-κB) is a transcription factor found in all animal cell types that plays a crucial role in immune and inflammatory responses [120]. As such, researchers have focused on understanding the NF-κB-related pathway, which presents a promising target for addressing a variety of pathological processes such as tumor progression, injury, and inflammation [121,122,123]. The NF-κB pathway has been implicated in regulating neuroinflammation following central nervous system diseases, making it a particularly important area of study [124]. Moreover, early research suggested, through kinase analysis, that NF-κB may play a critical role in ferroptosis [125]. Therefore, NF-κB holds the potential to emerge as a key focal point in the molecular mechanisms of both ferroptosis and neuroinflammation within the nervous system. A thorough understanding of the activation process and mechanism of NF-κB is essential for a more comprehensive prediction of the resulting damage and identification of potential intervention targets in central nervous system diseases.

Under normal physiological conditions, NF-κB remains inactive, typically bound to I-κB-α in the cytoplasm. However, when there is a change in the cytoplasmic environment or upon exposure to specific external stimuli, NF-κB and I-κB-α dissociate and translocate into the nucleus, where they regulate the transcription of specific genes, thereby activating NF-κB [103] (Figure 1). In the context of neuroinflammation, common cellular triggers that lead to NF-κB activation include reactive oxygen species (ROS), damage-associated molecular patterns (DAMPs), and lipopolysaccharides (LPSs) [103].

The generation of reactive oxygen species (ROS) is a key characteristic of cellular ferroptosis, with the disruption of cellular iron homeostasis leading to a sudden increase in intracellular ROS via the Fenton reaction [12]. In addition to instigating intracellular lipid peroxidation and oxidative stress, ROS can also activate the NF-κB pathway, thereby triggering further inflammatory cascades [126]. A recent study on ferroptosis demonstrated that reducing ROS-induced NF-κB activation and inhibiting the resulting neuroinflammatory response can be achieved through the use of the iron-chelating agent deferoxamine. The study further proposed that these effects may be mediated, at least in part, by the modulation of protein levels of p-NF-κBp65 [126]. This suggests that the regulation of neuroinflammation from the standpoint of iron homeostasis is, to some extent, dependent on NF-κB activity.

DAMPs serve as ubiquitous “warning signals” released from damaged or deceased cells, including neurons and glial cells within the brain [127]. One prominent DAMP in neuroinflammation is the high mobility group protein, HMGB1 [128]. In a study utilizing ischemia-reperfusion injury as a model, it was observed that IRI led to an increase in HMGB1 expression and upregulation of NF-κB p65 in brain tissue. Interestingly, the well-known ferroptosis inhibitor Fer-1 and its analogue Srs11-92 were able to reverse this phenomenon [129]. Notably, annexin A5, an annexin protein, has been shown to mitigate inflammation and ferroptosis following traumatic brain injury by targeting both the HMGB1/NF-κB pathway and the Nrf2 pathway [119].

Lipopolysaccharides have the ability to directly induce neuroinflammation and are frequently employed in constructing neuroinflammation models for numerous animal and cell culture experiments. Research has illustrated that lipopolysaccharide-induced neuroinflammation exerts its effects through the NF-κB pathway in cells such as microglia and neurons [130,131,132].

Within nerve cells, DAMPs and LPS function as ligands that bind to Toll-like receptor 4 (TLR4), initiating the activation of intracellular signaling pathways [132,133,134]. TLR4 triggers the activation of NF-κB via a splice protein known as myeloid differentiation primary response protein 88 (MyD88) [131,134] (Figure 1).

Upon entering the nucleus, NF-κB activates the NLRP3 inflammasome and caspase-1, consecutively. Subsequently, the activated caspase-1 converts the precursors of IL-1β and IL-18 into their active conformation [126]. The activation of the NF-κB pathway in innate immune cells within the brain ultimately elicits a shift towards a pro-inflammatory phenotype, ultimately resulting in the release of pro-inflammatory cytokines including TNF-α, IL-6, and IL-1β. These cytokines can exacerbate neuroinflammation [135,136]. Amongst these pro-inflammatory factors, IL-6 can contribute to ROS production [103], whereas TNF-α facilitates p65 binding factors to positively regulate NF-κB via nuclear signaling [137]. This amplifying effect may propagate inflammation throughout the brain.

The Fe^2+^/ROS/NF-κB inflammatory pathway serves as a crucial link between iron metabolism and cellular inflammation, offering a potential avenue for inhibiting neuroinflammation by regulating iron levels. Following brain injury, the administration of the iron-chelating agent DFO has been shown to elevate the protein level of p-NF-κBp65. This intervention not only diminishes the activation and infiltration of neutrophils and macrophages but also reverses the shift towards a pro-inflammatory phenotype in microglia-like cells, ultimately ameliorating cognitive dysfunction in mice [126]. Furthermore, the classical inhibitory pathway of ferroptosis, the GPX4 pathway, is intricately connected to the pro-inflammatory effects of NF-κB. Studies indicate that GPX4 can impede TNF-α-mediated NF-κB transcriptional activity [102]. The capacity of Saikosaponin B2 to suppress ferroptosis and neuroinflammation via the TLR4/NF-κB pathway relies on the presence of GPX4 [138].

NF-κB is indirectly modulated by SIRT1 and Nrf2, while it also influences the expression of SIRT1 and Nrf2 through its transcriptional products (Figure 1). The NF-κB transcription-induced inflammasome NLRP3 hampers SIRT1 synthesis [103]. Research indicates that the keap1-dependent pathway, which culminates in Nrf2 activation, is linked to the transcriptional activity of NF-κB p65 [139]. The interplay among the three intracellular regulatory molecules—SIRT1, Nrf2, and NF-κB—dictates the fate of neurons and glial cells. The cellular REDOX state may dictate the expression levels of these three proteins in response to external stimuli, with this equilibrium ultimately determining whether the nerve cell veers towards a pro-inflammatory and ferroptotic outcome.

#### 4.1.4. PTGS2/Cox-2

PTGS2, also known as prostaglandin-endoperoxide synthase 2, is a gene that regulates the synthesis of induced cyclooxygenase Cox-2. This physiological regulator catalyzes arachidonic acid (AA) to produce prostaglandins (PGs) and acts as a peroxidase [140,141]. Cox-2 is present in the human central nervous system under normal conditions and plays an important role in brain functions such as memory [142]. However, under pathological conditions, Cox-2 contributes to various neurodegenerative diseases and nerve injuries [142]. Elevated Cox-2 levels in cerebral stroke, traumatic brain injury, and neurodegeneration lead to pathological changes including blood–brain barrier breakdown and cerebral edema [143,144,145,146]. Furthermore, due to its role in lipid peroxidation during the catalytic process AA, Cox-2 serves as a lipid metabolic indicator of ferroptosis [147]. Additionally, Cox-2 is highly expressed in inflammation and correlates with inflammatory factors such as IL-1β, IL-6, and TNF-α [148].

Cox-2 primarily functions in acute inflammation, experiencing rapid upregulation following neuroinflammation and returning to baseline levels within a few hours [142]. The transcription factor NF-κB upregulates Cox-2 expression at the onset of inflammation by binding to Cox-2 promoters [149]. In models of LPS-induced neuroinflammation, NF-κB upregulation leads to elevated levels of Cox-2 and iNOS, initiating a cascade of acute inflammatory processes [150]. Given its crucial role in inflammation, Cox-2 inhibition has emerged as a significant therapeutic target for various inflammatory conditions [142]. Inhibiting Cox-2 has been demonstrated to suppress microglial activation, thereby reducing neuroinflammation, and downregulating Cox-2 also decreases NF-κB expression [151]. For example, in mouse models post-splenectomy, administration of the Cox-2 inhibitor meloxicam effectively mitigated neuroinflammation during the acute phase and alleviated cognitive dysfunction in the chronic phase [152].

Research into the impact of Cox-2 on ferroptosis extends beyond lipid peroxidation. Bioinformatics analysis has identified PTGS2 as a central gene in ferroptosis biology, closely associated with key players such as ACSL4 and GPX4 [153]. The regulation of Cox-2 is intertwined with ACSL4 expression, as evidenced by the concurrent decrease in Cox-2 content following ACSL4 gene knockdown [154]. Similarly, loss of GPX4 can result in increased PTGS2 expression [155]. Moreover, Cox-2 overexpression has been shown to diminish glutathione (GSH) levels through its interaction with GPX4 [156]. Consequently, Cox-2 expression can be modulated through the conventional ferroptosis inhibition pathway. It is evident that the expression of PTGS2 can be significantly elevated under the influence of ferroptosis-specific inducers like RSL3 and erastin, thereby impacting arachidonic acid metabolism and eicosanoid biosynthesis [135,140,157]. Conversely, Cox-2 inhibition can also influence the extent of ferroptosis. For instance, in a diffuse brain injury model, meloxicam treatment increased GSH levels and decreased malondialdehyde, an end product of ferroptosis, over a 48 h period [143]. Additionally, atorvastatin has been demonstrated to mitigate ferroptosis-induced myocardial injury through the PTGS2 pathway [158].

Particularly, research on the regulation of Cox-2 has revealed the existence of two types of non-coding RNAs (ncRNAs). One type is microRNA, a single-stranded RNA capable of binding to the 3’-untranslated region (UTR) of specific targeted mRNA [140]. The other type is long non-coding RNA (lncRNA), a long-stranded RNA that interferes with mRNA splicing and influences downstream gene expression. Importantly, lncRNAs, miRNAs, and downstream genes can form pathways that regulate proteins, known as competitive endogenous RNAs (ceRNAs) [159]. Based on targetscan, a database for predicting miRNA target genes, and bioinformatics analysis, ceRNAs have demonstrated exceptional performance in regulating PTGS2/Cox-2. In various systems, evidence has shown that miR-26a-5p and lncRNA Gm47283/miR-706 influence ferroptosis in bronchial epithelial cells in COPD and myocardial infarction via PTGS2, respectively [159,160]. Similarly, lncRNA-Cox2 serves as an inflammatory mediator, initiating a cascade of downstream inflammatory reactions by inducing transcription of NF-κB [161]. Numerous examples also illustrate how microRNAs and lncRNAs regulate PTGS2 to impact neuroinflammation and ferroptosis in brain disease models. For instance, miR-202-3p negatively regulates PTGS2 to attenuate neuroinflammation in IS [162]. In Alzheimer’s disease, lncMALAT1 negatively regulates the expression of Mir-125b-mediated PTGS2, while miR-103 promotes nerve recovery and growth by targeting PTGS2 [163,164]. Additionally, miR-137 from exosomes in endothelial progenitor cells can counteract the neuroprotective effect of SH-SY5Y (derived from neuroblasts) by inhibiting Cox-2 [165]. In rats, miR-194-5p targeting PTGS2 leads to nerve damage in temporal lobe epilepsy [166]. Studies have also found examples of miR-212-5p targeting PTGS2 to reverse ferroptosis in neurons in controlled cortical impact mouse models [140].

Indeed, the discovery of ceRNAs as upstream targets of PTGS2 that regulate Cox-2 protein expression presents exciting potential for microRNAs to modulate neuroinflammation and ferroptosis. As such, further research into the microRNA/PTGS2 pathway at the gene level is crucial. This will not only enhance our understanding of the interplay between neuroinflammation and ferroptosis in the central nervous system, but also pave the way for the development of corresponding interventional strategies for treating various clinical diseases.

#### 4.1.5. iNOS/NO•

Inducible nitric oxide synthase (iNOS) is an enzyme primarily responsible for catalyzing the synthesis of nitric oxide (NO). The metabolites of NO can generate nitroso salts, leading to oxidative reduction metabolic disorders [167]. iNOS has been detected in nerve damage caused by various factors and is closely associated with neuroinflammation and neuropathic pain [168]. The expression of iNOS has also been found in diverse central nervous system diseases, such as Alzheimer’s disease, Parkinson’s disease, ischemic brain injury, and traumatic brain injury [169,170,171,172]. However, the specific mechanisms through which iNOS influences the damage and long-term prognosis of these diseases are not yet fully understood, as compared to other molecules. This article aims to explore its relationship with ferroptosis and neuroinflammation in central nervous system diseases.

The neuroinflammation in the central nervous system caused by iNOS is closely linked to microglia. During traumatic neuroinflammation, microglia-produced TNF-α activates the NF-κB/iNOS pathway, leading to alterations in brain microcirculation function [172] (Figure 1). This type of nerve injury is primarily characterized by damage to the blood–brain barrier. In cerebral stroke, microglia rapidly produce iNOS upon cytokine stimulation [150]. Mechanistically, the regulation of iNOS mainly occurs at the transcriptional level and can be influenced by downstream molecules in the neuroinflammatory signaling network, consequently affecting upstream inflammatory factors. Following ischemic stroke, iNOS production enhances the expression of CD14, thereby activating the TLR4/NF-κB pathway and inducing the generation of inflammatory factors [171]. In neuroinflammatory models treated with LPS, microglia induce depression-like symptoms through the NLRP3/NF-κB/iNOS signaling pathway [173]. In the treatment of epilepsy, miconazole alleviates epilepsy symptoms by inhibiting both NF-κB and iNOS [174]. Thus, the interaction between NF-κB and iNOS plays a pivotal role in the development of central nervous system diseases.

The activity of iNOS and the resulting NO production significantly impact the phenotype of microglia, potentially serving as a critical factor in triggering neuroinflammation. LPS-treated microglia exhibit the M1 phenotype, accompanied by elevated levels of inflammatory factors and iNOS [175]. Studies indicate that the upregulation of iNOS/NO• and the rise of reactive oxygen species (ROS) due to disrupted iron metabolism lead to heightened resistance to ferroptosis in M1-polarized microglia, promoting a global shift toward the M1 phenotype [176,177]. This could explain the proinflammatory role of NO. Regarding the mechanism through which iNOS inhibits ferroptosis in microglia and macrophages, research has demonstrated that NO can independently regulate ferroptosis by suppressing the production of 15-HpETE-PE [178,179].

However, focusing solely on the effect of iNOS on microglia is one-sided, as numerous studies have demonstrated that iNOS and NO exert multiple effects on the ferroptosis of various cell phenotypes. For instance, in individuals who smoke cigarettes, NO produced by iNOS promotes ferroptosis in malignant mesothelioma (MM) cells through the production of peroxynitrite [180]. Furthermore, iNOS activation leads to the upregulation of ROS and RNS in triple-negative breast cancer cells, ultimately resulting in ferroptosis [181]. Interestingly, for the M1 and M2 phenotypes of macrophages, NO confers resistance to ferroptosis [182]. Within the current research landscape, NO has exhibited a protective effect against ferroptosis in certain immune cells, while demonstrating a sensitizing effect in other cells. Unraveling the underlying reasons behind these contrasting effects warrants further investigation.

Furthermore, contrary to the widely accepted notion of iNOS exerting detrimental effects on central nervous system diseases, emerging evidence suggests that iNOS may confer long-term protective effects on nerves [183]. This phenomenon could be attributed to the bidirectional impact of NO on lipid peroxidation. In the short term, its generation of peroxynitrite can mediate post-traumatic neuroinflammation and oxidative stress. However, owing to its ability to interact with other free radicals, NO also possesses unique antioxidant properties [183], thereby safeguarding nerves in the long run. Consequently, a comprehensive exploration of iNOS and NO warrants a closer examination of the temporal dynamics of disease and the intricate interplay between NO and other free radicals.

### 4.2. Cell Phenotype and Specific Structure

#### 4.2.1. Blood–Brain Barrier

The blood–brain barrier (BBB) plays a crucial role in facilitating communication between the brain and external substances. Any structural or functional abnormalities in the BBB can result in brain injury and cognitive dysfunction. Notably, numerous neurodegenerative diseases are marked by the impairment of the blood–brain barrier, such as stroke, traumatic brain injury (TBI), Alzheimer’s disease (AD), and Parkinson’s disease (PD) [184]. Therefore, it is imperative to gain a deeper understanding of the blood–brain barrier’s involvement in the pathological processes of central nervous system diseases.

**Figure 2:**
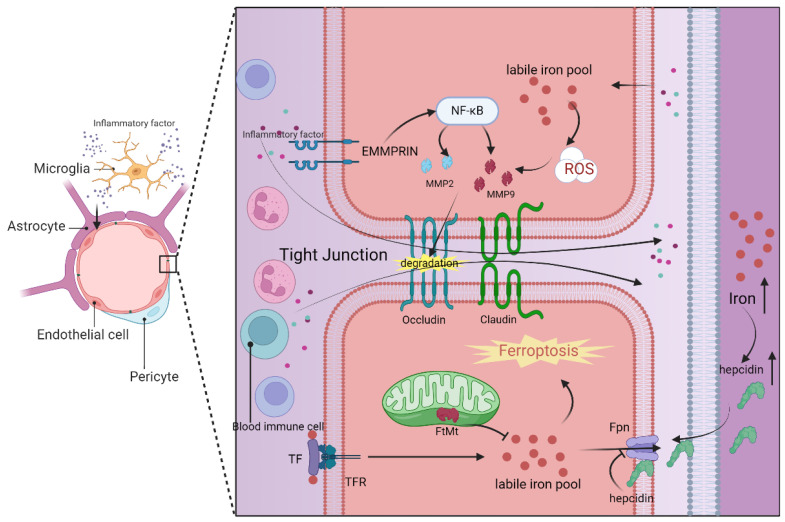
Structure of the blood–brain barrier and its intricate relationship with neuroinflammation and ferroptosis. Serving as a crucial barrier between brain capillaries and the brain parenchyma, the blood–brain barrier comprises brain endothelial cells, pericytes, and astrocytes, with a particular emphasis on the tight junctions between brain endothelial cells. In instances of brain inflammation, microglia are the primary instigators, releasing a plethora of inflammatory factors that disrupt the internal environment of various cell types within the blood–brain barrier, leading to elevated levels of iron and reactive oxygen species (ROS). Brain endothelial cells modulate the labile iron pool through mechanisms such as TF/TFR, FtMt, and FPN, while astrocyte-secreted hepcidin can impact the FPN activity of these endothelial cells. The accumulation of iron ultimately triggers ferroptosis in the brain endothelial cells. Moreover, EMMPRIN on the cell surface activates the NF-κB pathway, facilitating the secretion of MMP2/9, which in turn degrades tight junction proteins like occludin. The resultant ferroptosis of endothelial cells and the breakdown of tight junction proteins are primary contributors to the compromise of the blood–brain barrier integrity. This breach allows inflammatory factors from the bloodstream and peripheral immune cells to infiltrate the brain parenchyma, exacerbating neuroinflammation and eventual cell death within the brain parenchyma. Abbreviations: Fpn, ferroportin; FtMt, ferritin mitochondrial; MMP2, matrix Metallopeptidase 2; MMP9, matrix Metallopeptidase 9; NF-κB, nuclear factor-kappaB; ROS, reactive oxygen species; TF, transferrin; TFR, transferrin receptor. Solid arrows indicate promotion of expression or translocation. Inhibition arrows denote suppression of expression. Dotted arrows represent chemical names. Upward vertical arrows signify an increase in substance content. This figure was created with BioRender.com.

The blood–brain barrier is primarily composed of brain microvascular endothelial cells, pericytes, basement membrane, astrocyte terminal feet, and intercellular tight junction proteins, such as claudin-5 and ZO-1(Figure 2). Among these components, brain microvascular endothelial cells (BMECs) and tight junction structures (TJs) play the most crucial roles in determining the blood–brain barrier’s functionality [185,186]. Notably, BMECs lack transcellular fenestrae, which restricts the transcellular exchange pathway for substances. Additionally, the tight connection between BMECs minimizes intercellular substance exchange. When the blood–brain barrier is compromised, it often results in the infiltration of inflammatory mediators and toxic substances from the bloodstream into the brain [187]. This can occur through the cell death of brain microvascular endothelial cells, disruption of the tight junction structure, or a combination of both mechanisms, leading to a cascade of brain injuries.

Understanding the molecular mechanisms underlying blood–brain barrier damage and its consequences on the central nervous system is crucial due to the heavy reliance of the central nervous system on the physiological state of the neurovascular unit (NVU) in the brain [188]. Ferroptosis and neuroinflammation have emerged as significant mechanisms implicated in the progression and outcomes of blood–brain barrier injury during disease processes [189]. By comprehending these mechanisms, we can gain valuable insights into the pathogenesis of central nervous system diseases and potentially develop targeted therapeutic strategies.

Ferroptosis in brain microvascular endothelial cells plays a pivotal role in the compromised integrity of the blood–brain barrier. This intricate process encompasses a multitude of metabolic pathways and regulatory mechanisms, including iron metabolism, lipid metabolism, glutathione metabolism, and the GPX4 pathway [189]. Disruption in any of the components related to iron uptake, storage, or transport can elevate the labile iron pool in BMECs, resulting in heightened permeability of the blood–brain barrier. Consequently, BMECs transfer excess iron to neurons and glial cells in the brain via the transferrin and its receptor (TF/TFR1) mechanism [189] (Figure 2). Notably, mitochondrial ferritin (FtMt) in BMECs plays a crucial role in maintaining iron homeostasis by regulating iron storage [190] (Figure 2). Research has indicated that ferroportin 1 (FPN1), an iron transporter in BMECs, is regulated by hepcidin in astrocytes, underscoring the intricate intercellular regulation within the blood–brain barrier [191]. Furthermore, the use of the iron chelating agent DFO has been shown to reduce the labile iron pool in BMECs and protect the blood–brain barrier through the HIF2α-Ve-Cadherin pathway [192]. Additionally, elevated levels of lipid peroxidation products, such as MDA and 4HNE, have been observed in association with blood–brain barrier disruption, concomitant with depleted GSH levels, emphasizing the potential significance of REDOX regulation in preserving the integrity of the blood–brain barrier [189].

In addition to BMECs, other components of the blood–brain barrier have also been implicated in ferroptosis and its associated metabolic pathways. Notably, in the context of Alzheimer’s disease treatment, Aβ1-40 has been found to facilitate iron overload and lipid peroxidation in pericytes, thereby inducing blood–brain barrier impairment through pericyte-mediated ferroptosis [193]. Moreover, lipid peroxidation can have an effect on the blood–brain barrier by downregulating tight junction proteins, further compromising its integrity [184].

In the study of different central nervous system diseases, researchers have discovered that brain microvascular endothelial cells exhibit heightened sensitivity to ferroptosis compared to other cell types [194]. In various models of brain injury resulting from cerebral ischemia and hypoxia, Alzheimer’s disease, traumatic brain injury, and similar conditions, ferroptosis triggered by BMECs has emerged as a crucial factor contributing to the loss of blood–brain barrier integrity [194,195,196]. Considering the interplay between blood–brain barrier cells and the impact of tight junction proteins affected by metabolites associated with cell ferroptosis, it can be inferred that cell ferroptosis represents a significant underlying cause of blood–brain barrier disruption.

Most central nervous system diseases are associated with neuroinflammatory cascades, and the relationship between cerebral neuroinflammation and the blood–brain barrier is bidirectional. Neuroinflammation contributes to an increase in blood–brain barrier permeability due to various factors, while dysfunction of the blood–brain barrier leads to an amplification of the neuroinflammatory response [52]. At the core of this positive feedback loop is microglia, which can initiate inflammatory pathways following brain damage from diverse causes, impact the structure of BMECs, and disrupt the tight junctions of the blood–brain barrier through the release of inflammatory cytokines. Once the cell–cell connections of the blood–brain barrier are compromised, the infiltration of peripheral immune cells and exogenous neurotoxic substances further stimulate microglia [52,197]. This sequence of events encompasses several classic inflammatory manifestations, including the release of TNF-α, IL-6, and IL-1β, as well as the transition of microglia from the M2 to M1 phenotype [198,199]. In a particular study, researchers utilized lipopolysaccharide-induced neuroinflammation to examine the microstructure of the blood–brain barrier. Their findings revealed morphological changes in brain endothelial cells (BEC), such as increased vesicles, plasma membrane shrinkage, mitochondrial abnormalities, and extracellular expulsion. Additionally, notable oxidative stress characteristics were observed in BECs [200]. Whether these morphological and functional alterations are linked to the ferroptosis of BECs warrants further investigation.

In the progression of neuroinflammation, the destruction of the blood–brain barrier leads to the invasion of peripheral circulating immune cells and other harmful substances, which is a significant mechanism for exacerbating brain injury (Figure 2). First, the chemokines and cytokines released by peripheral immune cells can directly breach the blood–brain barrier and activate the inflammatory response in the brain parenchyma [200]. Secondly, the infiltration of the peripheral immune cell barrier into the brain can lead to damage of the original neurovascular unit (NVU) [52]. Finally, the proinflammatory phenotypic changes in microglia are also directly or indirectly associated with the infiltration of peripheral cells [197].

Indeed, matrix metalloproteinases (MMPs), particularly MMP-2 and MMP-9, play a crucial role in the mechanism of blood–brain barrier damage [201]. These MMPs can be induced by extracellular matrix metalloproteinase inducer (EMMPRIN). Elevated levels of MMP-2/9 can lead to blood–brain barrier dysfunction by degrading tight junction proteins like ZO-1 and occludin that maintain cellular integrity [202]. There are various regulatory mechanisms for MMP-2/9. Evidence suggests that lipid peroxidation in endothelial cells can upregulate MMP-2/9, leading to the breakdown of occludin [184]. Additionally, activated microglia can stimulate MMP-2/9 by increasing EMMPRIN expression through specific signaling pathways [189,202] (Figure 2). This information highlights how both the process of ferroptosis in cells and neuroinflammatory manifestations can contribute to blood–brain barrier damage through MMPs. EMMPRIN and MMP-2/9 may serve as important links connecting neuroinflammation and cellular ferroptosis in the context of the blood–brain barrier, making them potential targets for the treatment of central nervous system diseases.

The molecular mechanisms underlying the onset and progression of blood–brain barrier (BBB) injury are intricate, given its crucial role in separating and connecting the central nervous system with the peripheral blood circulation. Following the emergence of central nervous system diseases, neuroinflammation and the occurrence of ferroptosis in brain microvascular endothelial cells (BMECs) and other cell types contribute to the breakdown of the BBB’s essential structure. This heightened permeability of the BBB not only fosters a cycle of neuroinflammatory responses but also gives rise to diverse forms of neurovascular unit (NVU) cell death in the brain. In essence, ferroptosis and neuroinflammation can act as both instigators and outcomes of BBB injury, respectively. Pertinent inquiries arise: (a) What specific mechanisms underpin cell ferroptosis in causing BBB damage? (b) Are the phenotypic changes in BMECs, driven by neuroinflammation subsequent to central nervous system diseases, linked to its ferroptosis mechanism? (c) Is the induced NVU cell death following BBB injury directly associated with alterations in BMECs and tight junctions (TJs)?

#### 4.2.2. Microglia

Microglia are a distinct type of glial cell found in the central nervous system, primarily localized around neurons and the cerebrovasculature, and their population is relatively small compared to other glial cells. Under normal, homeostatic conditions, resting microglia serve essential physiological functions such as promoting nerve growth and constructing synapses [203]. However, when the brain experiences injury or neurodegeneration, this delicate balance within the central nervous system is disrupted, leading to morphological and functional changes in microglia as they become activated [48]. The activated microglia exhibit diverse characteristics, and in order to facilitate the study of their function, researchers have traditionally classified their activation morphology into the pro-inflammatory M1 phenotype and the anti-inflammatory M2 phenotype [204]. This marked functional shift may be attributed to differing metabolic profiles in the two phenotypes [204]. Throughout the progression of central nervous system diseases, certain pathogenic molecules can induce the transformation of microglia into the M1 phenotype. These pathogenic molecules include pathogen-associated molecular patterns (PAMPs) such as lipopolysaccharides, cytokines like IFNγ and GM-CSF, or DAMPs such as HMGB-1 or alpha-SYN [203].

Significantly, ferroptosis is intricately linked to phenotypic changes in microglia. Firstly, iron metabolism in microglia plays a critical role in influencing its phenotypic shifts. Research indicates that during the acute phase of Alzheimer’s disease or LPS-induced neuroinflammation, the upregulation of divalent metal transporter 1 (DMT1) on the microglial cell membrane results in iron overload and M1 polarization in microglia [136,205]. The activation of M1 phenotype microglia leads to the release of inflammatory factors such as TNF-α, IL-1β, and IL-6, exacerbating neuroinflammation [102,138]. The classical iron chelating agent DFO has been shown to help maintain iron homeostasis and partially reverse M1 polarization of microglia [126].

Secondly, lipid peroxidation can induce the polarization of microglia towards the M1 phenotype, thereby triggering microglia-mediated neuroinflammation [206]. Notably, the signature molecule of ferroptosis, Acyl-CoA synthetase long-chain family member 4 (ACSL4), which regulates polyunsaturated fatty acids, has been implicated in enhancing the release of inflammatory factors in microglia during stroke. The use of the ferroptosis inhibitor liproxstatin-1 has demonstrated efficacy in reducing neuroinflammation resulting from increased ACSL4 expression [26]. However, while ACSL4 in microglia does contribute to the production of inflammatory cytokines, studies suggest that this mechanism may be independent of ferroptosis. Data indicate that ACSL4 influences neuroinflammation by affecting vestigial-like family member 4 (VGLL4), with no significant changes in lipid peroxidation and cell-ferroptosis-related indicators observed following ACSL4 knockdown [206].

Finally, REDOX metabolism and related glutathione signaling pathways play a crucial role in influencing phenotypic changes in microglia. Reduced levels of GPX4 in the brain after injury result in the upregulation of reactive oxygen species (ROS), which in turn enhances the pro-inflammatory response of microglia through the NF-κB signaling pathway [126,135]. Studies involving mice with GPX4 gene knockout have demonstrated increased microglial activation and upregulation of neuroinflammatory markers [127].

Current research on the mechanism of ferroptosis on microglia phenotype has certain limitations. This mechanism may be related to the varying levels of inducible nitric oxide synthase (iNOS) in different cell phenotypes [175]. Regardless of whether it is the M1 or M2 phenotype, microglia can resist ferroptosis through the iNOS/nitric oxide (NO•) pathway. However, the expression of iNOS in microglia of the M2 phenotype is relatively low, which leads to the death of the M2 phenotype under conditions of iron overload [176]. Following subarachnoid hemorrhage, the administration of L-NIL, an inhibitor of iNOS, can reverse the number of M1 phenotypes in microglia and mitigate the extent of damage caused by SAH [177].

In addition, the inflammatory effect of microglia is reflected in its role in mediating the death of other nerve cells within the central nervous system. Microglia can disrupt the blood–brain barrier by releasing pro-inflammatory factors that damage brain microvascular endothelial cells and tight junction proteins [52]. Microglia activated prior to astrocytes can influence astrocyte A1/A2 phenotypes through pro-inflammatory/anti-inflammatory factors, thereby influencing astrocyte outcomes [207]. In mice with Parkinson’s disease, microglia can induce ferroptosis in neuronal cells [107].

Microglia, as the core cells of central nervous system inflammation, play a crucial role in mediating and perpetuating inflammation. They can be activated and migrate in response to chemokines and cytokines released during inflammation, subsequently releasing pro-inflammatory factors that contribute to the inflammatory cascade. Consequently, cell death and phenotypic changes within microglia themselves become significant factors in the progression of inflammation. Several studies have demonstrated that the sensitivity of microglia to ferroptosis, depending on their phenotypes, influences their polarization during central nervous system inflammation, thereby exerting a comprehensive impact on neuroinflammation. Furthermore, as cells undergoing ferroptosis release DAMPs that promote neuroinflammation [127], the involvement of ferroptosis in other related cells affected by microglia contributes to the pathological changes observed in central nervous system diseases. However, the mechanisms through which microglia induce ferroptosis in themselves and other nerve cells warrant further investigation.

#### 4.2.3. Astrocytes

Astrocytes, the largest glial cell type, are ubiquitously present across mammalian species. These cells play a pivotal role within the central nervous system, where their primary functions span from maintaining ion homeostasis, metabolizing neurotransmitters, and providing neurotrophic support to contributing to the integrity of the blood–brain barrier [53,208,209,210]. Recent years have seen a surge in interest regarding the dynamic interaction between astrocytes and neurons. Beyond merely creating an optimal physiological milieu for neuronal function, astrocytes emerge as autonomous information-processing units that significantly influence the central nervous system’s overarching functionality [211].

Astrocyte lesions play a critical role in a wide array of central nervous system diseases, including traumatic brain injury, neurodegenerative disorders, and cerebrovascular diseases. The pathophysiological changes observed in astrocytes during these conditions typically include morphological atrophy, various modes of cell death, and the proliferation of reactive astrocytes [212]. Among these, reactive astrocytosis has garnered significant attention. Initially, much like microglia, reactive astrocytes were categorized into neurotoxic (A1) and neuroprotective (A2) types, reflecting their dual roles in neural environments [204]. However, emerging perspectives suggest that astrocytes’ response to disease and trauma is predominantly adaptive, thus primarily serving a neuroprotective function [212]. This shift in understanding underscores the importance of focusing research on the functional phenotypes of reactive glial cells rather than a binary classification, offering new insights into astrocytes’ involvement in neuroinflammation.

Current research indicates that astrocytes undergo phenotypic transformations that play a crucial role in modulating neuroinflammation, leading to the production of various inflammatory cytokines and cytotoxic reactive oxygen species (ROS) [49]. Interestingly, astrocytes can generate antioxidant enzymes under pathological conditions to mitigate oxidative stress [49]. During inflammation, astrocytes’ regulation of mitochondrial functions, ROS, and antioxidant systems is pivotal in controlling their susceptibility to ferroptosis [213]. Building on this understanding and the intricate material exchanges between astrocytes and neurons, we delve into the connection between astrocyte-driven neuroinflammation and ferroptosis, highlighting the complex interplay that influences disease progression and potential therapeutic targets.

During the neuroinflammation of the telencephalon, astrocytes become activated, identifiable by specific biomarkers such as GFAP, S100β, and CD81 [49,214]. The functionality of astrocytes is intricately linked to that of microglia, with various proinflammatory and anti-inflammatory cytokines and chemokines serving as crucial mediators of their interaction. Both cell types are capable of influencing the other’s phenotype through the release of factors like TNF-α, which promotes inflammation, or TGF-β, which dampens it, thereby either exacerbating or mitigating the inflammatory response [49,204]. Furthermore, activated astrocytes can produce matrix metalloproteinases (MMPs), compromising the blood–brain barrier and facilitating the influx of peripheral inflammatory cells into the central nervous system, thereby intensifying the inflammatory milieu within the brain [49]. 

The release of pro-inflammatory and anti-inflammatory mediators during inflammation, coupled with oxidative stress-related enzymes, triggers the activation of intracellular receptors and signaling pathways. Notably, NADPH oxidase 4 (NOX4) in astrocytes catalyzes the production of reactive oxygen species (ROS), further promoting neuroinflammation and mitochondrial damage [215]. This ROS-induced activity leads to lipid peroxidation within cells, elevating levels of 4HNE and MDA and initiating ferroptosis in astrocytes [213,216]. The activation of ROS also correlates with increased concentrations of IL-1β, IL-6, TNF-α, and other inflammatory cytokines, suggesting a temporal overlap between the induction of astrocyte inflammation and ferroptosis. The upregulation of glutathione peroxidase 4 in astrocytes acts as a defense mechanism against oxidative stress-induced ferroptosis [49].

Moreover, the proinflammatory phenotype of astrocytes appears to be closely associated with the NF-κB signaling pathway. The interplay between Nrf2 and NF-κB pathways is central to the molecular mechanisms underlying neurological diseases. Nrf2 can mitigate the expression of reactive astrocytes and reduce neuroinflammation and ferroptosis by inhibiting NF-κB [217,218]. The regulator of G protein signaling 5 (RGS5) can induce neuroinflammation via the tumor necrosis factor receptor (TNFR) signaling pathway, contributing to neurodegeneration in conditions like Parkinson’s disease, with TLR4 mediating downstream factors of this pathway and regulating NF-κB activation through nuclear translocation [131,219]. Experiments treating angiotensin II (Ang II)-stimulated astrocytes with the ferroptosis-specific inhibitor ferrostatin-1 have shown concurrent alterations in astrocyte markers, inflammatory factors, and ferroptosis-related proteins [58]. Therefore, there may be a common regulatory approach between astrocyte-induced neuroinflammation and cellular ferroptosis.

Furthermore, the process of inflammatory induction in astrocytes can trigger ferroptosis in neurons by altering the internal milieu of the central nervous system. Chemokines derived from astrocyte activation, such as CXCL10/CXCR3, and the inflammatory cytokine IL-6 can alter the expression of neuronal proteins, including Fpn, leading to an increase in intracellular iron levels and ultimately resulting in neuronal ferroptosis [29,220]. However, recent studies have indicated that the regulation of ferroptosis in neurons by astrocytes is complex. Astrocytes may leverage the protective potential of Nrf2 to counter ferroptosis in neurons by releasing exosomes [221]. 

#### 4.2.4. Neutrophils and NETs

Neutrophils, originating from the bone marrow, are widely distributed in the bloodstream and play a crucial role in acute inflammation, being the most prevalent cells during its initial stages. Evidence suggests that the Neutrophil to Lymphocyte Ratio (NLR) is associated with prognosis in certain central nervous system diseases [222,223,224]. However, the physiological blood–brain barrier presents a challenge for neutrophil entry into the telencephalon. Consequently, the contribution of peripheral cells to central nervous system diseases is often underestimated. Nevertheless, various pathological conditions can compromise the blood–brain barrier, eventually allowing neutrophil infiltration. The interplay between neutrophils and blood–brain barrier integrity is an unavoidable subject in central nervous system diseases. Both systemic circulation and inflammatory factors in the brain milieu can contribute to blood–brain barrier disruption [225]. Under pathological conditions, chemokines and cytokines can drive neutrophil aggregation towards the blood–brain barrier, further exacerbating its integrity [226]. Once the blood–brain barrier is breached, neutrophils can infiltrate the brain, triggering neuroinflammation and damage to the nervous system.

Recent advances have reshaped the understanding of neutrophils, highlighting their diverse phenotypes and functions, contrary to the prior notion of their homogeneity [227]. Different neutrophil phenotypes play distinct roles in brain neuroinflammation [228]. Studies on neutrophil aging indicate that prolonged neutrophil survival amplifies their pro-inflammatory impact on the brain [229]. Over the years, research on Neutrophil Extracellular Traps (NETs) in neurological disorders has surged. NETs represent the unique response of mature neutrophils to trauma and pathogens, forming a meshwork structure for self-destruction [230]. Co-culturing of NETs with nerve cells has demonstrated their ability to provoke nerve cell inflammation. Additionally, ferroptosis in neutrophils contributes to NET formation [231]. The inhibition of NET formation by ferroptosis inhibitor ferrostatin-1 underscores the intimate link between ferroptosis and NET generation in neutrophils [231]. Nonetheless, there is a lack of in vivo evidence of neutrophil ferroptosis post-infiltration into brain tissue through the compromised blood–brain barrier, triggering NET release and exacerbating neuroinflammation. Studies have shown that NETs modulate ferroptosis in alveolar epithelium and breast cancer cell death through NF-κB-related pathways [232,233]. The intricate interplay among reactive oxygen species (ROS), NF-κB, and other inflammatory factors in inducing neuroinflammation and ferroptosis presents a compelling avenue for exploring the processes and molecular mechanisms of NET-driven ferroptosis and neuroinflammation in the brain.

Furthermore, both inflammatory cells and ferroptotic cells can release hypermigratory histone box 1 (HMGB1), a protein that plays a crucial role in neutrophil recruitment [234]. Based on the aforementioned discoveries, we propose several hypotheses: In certain central nervous system disorders, such as degenerative diseases and acute-phase conditions like trauma, there may exist a detrimental cycle involving intracerebral nerve cells, peripheral neutrophils, and intracerebral nerve cells. The release of DAMPs and inflammatory factors during neuroinflammation of brain cells can lead to neutrophil recruitment and disruption of the blood–brain barrier. Consequently, neutrophil infiltration into brain tissue occurs, triggering their ferroptosis. Following ferroptosis, neutrophils release NETs, which can further exacerbate the inflammatory response and neuronal death through interactions with neurons.

#### 4.2.5. T Cells

Lymphocytes are pivotal in orchestrating immune responses within the body. Based on their morphology and function, they can be classified into B lymphocytes, T lymphocytes, and natural killer (NK) cells. In certain neurological conditions that incite chronic neuroinflammation, both B and T cells have been observed to infiltrate the cerebrospinal fluid, meninges, blood–brain barrier, and brain parenchyma [51,235,236,237]. Nevertheless, extensive research indicates that T lymphocytes predominantly contribute to the pathogenesis of central nervous system diseases. B cells, on the other hand, play a crucial role in maintaining the microenvironment and activating and sustaining the pro-inflammatory function of T cells [235,236].

It is important to note that chemokines play a crucial role in the infiltration and proliferation of T cells and B cells within the central nervous system. Chemokines can be classified into four different subgroups: CXC, CC, CX3C, and XC [238]. Lymphocytes express various chemokine receptors, which enable them to access different sites via the chemokine ligand/receptor axis when chronic neuroinflammation occurs [239].

T lymphocytes are categorized into cytotoxic T cells (Tc cells), helper T cells (Th cells), and regulatory T cells (Treg cells) based on their functions. These T lymphocytes play a significant role in immune-related and chronic infectious neurological diseases by releasing cytokines. Helper T cells, specifically Th1 and Th17 cells, are strongly associated with neuroinflammation in the central nervous system. Research has demonstrated that CNS astrocytes respond to cytokines released during Th1 and Th17 cell activation [240]. Th1 cells can secrete IFN-γ, which promotes the infiltration of T cells into the spinal cord. Meanwhile, astrocytes partially inhibit T cell infiltration through the VCAM-1 receptor [240]. In the brain, the invasion of Th cells involves antigen-presenting cells (APCs) such as boundary-associated macrophages (BAMs), classical dendritic cells (cDCs), and B cells [237]. Activation of Th1 and Th17 cells plays a critical role in promoting neuroinflammation by T cells. Jinyuan et al. discovered that in multiple sclerosis, neurons undergoing ferroptosis stimulate the activation of T cell receptor signaling pathways through the secretion of various related molecules, leading to the activation of Th1 and Th17 cells and the release of cytokines like IFN-γ and IL-17 [241].

CD8+ T cells are crucial cytotoxic T cells that have destructive effects on both brain parenchyma and blood vessels. In chronic neuroinflammation, CD8+ T cells can infiltrate and destroy blood vessels. In pathological models of cerebral malaria and Susac syndrome, CD8+ T cells can damage cerebral vascular endothelial cells and decrease tight junction protein expression via adhesion molecules such as VCAM-1 [242]. The breakdown of the blood–brain barrier by CD8+ T cells ultimately leads to their infiltration into the brain parenchyma [51]. Activated CD8+ T cells play a vital role in inducing ferroptosis in neurons, which is confirmed by upregulation of related proteins such as TFR1 and ACSL4 in ECM brain models [243]. Th cell activation by neuronal ferroptosis may locally exacerbate the “vicious circle” of neuroinflammation [51]. Additionally, the infiltration of CD8+ T cells in tau transgenic mice may activate microglia and astrocytes on the surface, leading to further aggravation of neuroinflammation in the brain [244]. In contrast, Treg cells often display neuroprotective effects, and their proliferation can be promoted by IL-2 secreted by helper T cells [245]. Depleting Treg cells in cases of brain injury is not favorable for recovery.

Peripheral immune cells represent a promising area of research in the context of central nervous system inflammation. While previous studies of CNS diseases have primarily focused on the interaction between neurons and glial cells in situ, the significance of peripheral immune cells in the blood–brain barrier and brain parenchyma cannot be overlooked. Particularly in certain chronic neuroinflammation disease models, there is evidence suggesting that neuronal ferroptosis may both result from and contribute to the aggregation and activation of T cells. The infiltration of immune cells exacerbates the disruption of the nervous system’s physiological equilibrium and intensifies neuroinflammation. The quantity and function of lymphocytes in numerous central nervous system disease models warrant further investigation.

## 5. Conclusions

This review comprehensively summarizes the molecular mechanisms and cellular responses linked to ferroptosis and neuroinflammation in central neurological disorders. Our literature search revealed a potential interplay between the metabolites of ferroptosis and neuroinflammation, with each potentially acting as the initiator of the other process. While the detailed pathogenesis varies, various central neurological disorders involve immune cell activation in the brain, disruption of the blood–brain barrier, intracranial iron overload, and peroxidation. These changes underscore the close correlation between ferroptosis and neuroinflammation in the spatial and temporal distribution of each disease. Furthermore, we focused on elucidating the specific mechanisms involving SIRT1, Nrf2, NF-κB, Cox-2, and iNOS/NO• molecules upstream and downstream of each other in central neurological disorders, highlighting their connections with ferroptosis and neuroinflammation. However, the relationships between these molecules are intricate, with many intermediate processes still not fully understood. Moreover, the varying content of these regulatory molecules in different nervous system cells significantly influences the susceptibility of different cell phenotypes to ferroptosis and neuroinflammation. Future research on these mechanisms could delve deeper into the following aspects: a. Intermediate processes linking these associated molecules. b. Variances in the impact of different molecules over time and across various cell phenotypes. c. The influence of peripheral immune cells on the central nervous system environment.

## 6. Futures Perspectives

Given the intricate structure of the human brain and the diverse functions of nerve cells, the treatment options for many central neurological disorders are currently limited. Consequently, researchers are concentrating on exploring new molecular mechanisms underlying these diseases. Ferroptosis, identified as a specific form of cell death, has been implicated in various neurological disorders. By elucidating the interactive signaling pathways and critical regulatory molecules connecting ferroptosis and neuroinflammation, we have broadened the spectrum of potential therapeutic targets aimed at mitigating neurological damage in central nervous system disorders. For instance, resveratrol, which modulates SIRT1, shows promise as a viable treatment for a range of neuroinflammatory conditions [93]. Undoubtedly, further endeavors are imperative to translate anti-ferroptosis or anti-inflammatory drugs into clinical practice. Apart from conducting phased animal studies and clinical trials, enhancing the integration between the two pathways is equally pivotal.

## References

[1] Sauve F., Nampoothiri S., Clarke S.A., Fernandois D., Ferreira Coelho C.F., Dewisme J., Mills E.G., Ternier G., Cotellessa L., Iglesias-Garcia C. (2023). Long-COVID cognitive impairments and reproductive hormone deficits in men may stem from GnRH neuronal death. EBioMedicine.

[2] Choi B.Y., Hong D.K., Kang B.S., Lee S.H., Choi S., Kim H.J., Lee S.M., Suh S.W. (2023). Engineered Mesenchymal Stem Cells Over-Expressing BDNF Protect the Brain from Traumatic Brain Injury-Induced Neuronal Death, Neurological Deficits, and Cognitive Impairments. Pharmaceuticals.

[3] Pang K., Jiang R., Zhang W., Yang Z., Li L.L., Shimozawa M., Tambaro S., Mayer J., Zhang B., Li M. (2022). An App knock-in rat model for Alzheimer’s disease exhibiting Abeta and tau pathologies, neuronal death and cognitive impairments. Cell Res..

[4] Dixon S.J., Lemberg K.M., Lamprecht M.R., Skouta R., Zaitsev E.M., Gleason C.E., Patel D.N., Bauer A.J., Cantley A.M., Yang W.S. (2012). Ferroptosis: An iron-dependent form of nonapoptotic cell death. Cell.

[5] Chen D., Fan Z., Rauh M., Buchfelder M., Eyupoglu I.Y., Savaskan N. (2017). ATF4 promotes angiogenesis and neuronal cell death and confers ferroptosis in a xCT-dependent manner. Oncogene.

[6] Zhang Y., Shaabani S., Vowinkel K., Trombetta-Lima M., Sabogal-Guaqueta A.M., Chen T., Hoekstra J., Lembeck J., Schmidt M., Decher N. (2024). Novel SK channel positive modulators prevent ferroptosis and excitotoxicity in neuronal cells. Biomed. Pharmacother..

[7] Cagnin A., Gerhard A., Banati R.B. (2002). In vivo imaging of neuroinflammation. Eur. Neuropsychopharmacol..

[8] Adrian M., Weber M., Tsai M.C., Glock C., Kahn O.I., Phu L., Cheung T.K., Meilandt W.J., Rose C.M., Hoogenraad C.C. (2023). Polarized microtubule remodeling transforms the morphology of reactive microglia and drives cytokine release. Nat. Commun..

[9] Jiang W., Wang Y., Sun W., Zhang M. (2017). Morin Suppresses Astrocyte Activation and Regulates Cytokine Release in Bone Cancer Pain Rat Models. Phytother. Res..

[10] Fang D., Guo S., Wei B., Liu W., Li G., Li X., Liu J., Jin L., Duan C. (2024). Nrf-2 modulates excitability of hippocampal neurons by regulating ferroptosis and neuroinflammation after subarachnoid hemorrhage in rats. Brain Res. Bull..

[11] Nemeth E., Rivera S., Gabayan V., Keller C., Taudorf S., Pedersen B.K., Ganz T. (2004). IL-6 mediates hypoferremia of inflammation by inducing the synthesis of the iron regulatory hormone hepcidin. J. Clin. Investig..

[12] Dong B., Jiang Y., Shi B., Zhang Z., Zhang Z. (2024). Selenomethionine alleviates decabromodiphenyl ether-induced oxidative stress and ferroptosis via the NRF2/GPX4 pathway in the chicken brain. J. Hazard. Mater..

[13] Mumbauer S., Pascual J., Kolotuev I., Hamaratoglu F. (2019). Ferritin heavy chain protects the developing wing from reactive oxygen species and ferroptosis. PLoS Genet..

[14] Luo C., Xu W., Tang X., Liu X., Cheng Y., Wu Y., Xie Z., Wu X., He X., Wang Q. (2022). Canonical Wnt signaling works downstream of iron overload to prevent ferroptosis from damaging osteoblast differentiation. Free Radic. Biol. Med..

[15] Hu W., Zhou C., Jing Q., Li Y., Yang J., Yang C., Wang L., Hu J., Li H., Wang H. (2021). FTH promotes the proliferation and renders the HCC cells specifically resist to ferroptosis by maintaining iron homeostasis. Cancer Cell Int..

[16] Bao X., Luo X., Bai X., Lv Y., Weng X., Zhang S., Leng Y., Huang J., Dai X., Wang Y. (2023). Cigarette tar mediates macrophage ferroptosis in atherosclerosis through the hepcidin/FPN/SLC7A11 signaling pathway. Free Radic. Biol. Med..

[17] Yang S., Zhang T., Ge Y., Cheng Y., Yin L., Pu Y., Chen Z., Liang G. (2023). Ferritinophagy Mediated by Oxidative Stress-Driven Mitochondrial Damage Is Involved in the Polystyrene Nanoparticles-Induced Ferroptosis of Lung Injury. ACS Nano.

[18] Tuo Q.Z., Liu Y., Xiang Z., Yan H.F., Zou T., Shu Y., Ding X.L., Zou J.J., Xu S., Tang F. (2022). Thrombin induces ACSL4-dependent ferroptosis during cerebral ischemia/reperfusion. Signal Transduct. Target. Ther..

[19] Wu C., Du M., Yu R., Cheng Y., Wu B., Fu J., Tan W., Zhou Q., Balawi E., Liao Z.B. (2022). A novel mechanism linking ferroptosis and endoplasmic reticulum stress via the circPtpn14/miR-351-5p/5-LOX signaling in melatonin-mediated treatment of traumatic brain injury. Free Radic. Biol. Med..

[20] Zhou Q., Meng Y., Li D., Yao L., Le J., Liu Y., Sun Y., Zeng F., Chen X., Deng G. (2024). Ferroptosis in cancer: From molecular mechanisms to therapeutic strategies. Signal Transduct. Target. Ther..

[21] Doll S., Freitas F.P., Shah R., Aldrovandi M., da Silva M.C., Ingold I., Goya Grocin A., Xavier da Silva T.N., Panzilius E., Scheel C.H. (2019). FSP1 is a glutathione-independent ferroptosis suppressor. Nature.

[22] Mao C., Liu X., Zhang Y., Lei G., Yan Y., Lee H., Koppula P., Wu S., Zhuang L., Fang B. (2021). DHODH-mediated ferroptosis defence is a targetable vulnerability in cancer. Nature.

[23] Hu Q., Wei W., Wu D., Huang F., Li M., Li W., Yin J., Peng Y., Lu Y., Zhao Q. (2022). Blockade of GCH1/BH4 Axis Activates Ferritinophagy to Mitigate the Resistance of Colorectal Cancer to Erastin-Induced Ferroptosis. Front. Cell Dev. Biol..

[24] Jacquemyn J., Ralhan I., Ioannou M.S. (2024). Driving factors of neuronal ferroptosis. Trends Cell Biol..

[25] Selim M.H., Ratan R.R. (2004). The role of iron neurotoxicity in ischemic stroke. Ageing Res. Rev..

[26] Cui Y., Zhang Y., Zhao X., Shao L., Liu G., Sun C., Xu R., Zhang Z. (2021). ACSL4 exacerbates ischemic stroke by promoting ferroptosis-induced brain injury and neuroinflammation. Brain Behav. Immun..

[27] Ashraf A., Jeandriens J., Parkes H.G., So P.W. (2020). Iron dyshomeostasis, lipid peroxidation and perturbed expression of cystine/glutamate antiporter in Alzheimer’s disease: Evidence of ferroptosis. Redox Biol..

[28] Ding X.-S., Gao L., Han Z., Eleuteri S., Shi W., Shen Y., Song Z.-Y., Su M., Yang Q., Qu Y. (2023). Ferroptosis in Parkinson’s disease: Molecular mechanisms and therapeutic potential. Ageing Res. Rev..

[29] Liang P., Zhang X., Zhang Y., Wu Y., Song Y., Wang X., Chen T., Liu W., Peng B., Yin J. (2023). Neurotoxic A1 astrocytes promote neuronal ferroptosis via CXCL10/CXCR3 axis in epilepsy. Free Radic. Biol. Med..

[30] Xie B.S., Wang Y.Q., Lin Y., Mao Q., Feng J.F., Gao G.Y., Jiang J.Y. (2019). Inhibition of ferroptosis attenuates tissue damage and improves long-term outcomes after traumatic brain injury in mice. CNS Neurosci. Ther..

[31] Schroder N., Figueiredo L.S., de Lima M.N. (2013). Role of brain iron accumulation in cognitive dysfunction: Evidence from animal models and human studies. J. Alzheimers Dis..

[32] Fredriksson A., Schröder N., Eriksson P., Izquierdo I., Archer T. (1999). Neonatal iron exposure induces neurobehavioural dysfunctions in adult mice. Toxicol. Appl. Pharm..

[33] Mei H., Wu D., Yong Z., Cao Y., Chang Y., Liang J., Jiang X., Xu H., Yang J., Shi X. (2024). PM(2.5) exposure exacerbates seizure symptoms and cognitive dysfunction by disrupting iron metabolism and the Nrf2-mediated ferroptosis pathway. Sci. Total Environ..

[34] Xie R., Zhao W., Lowe S., Bentley R., Hu G., Mei H., Jiang X., Sun C., Wu Y., Liu Y. (2022). Quercetin alleviates kainic acid-induced seizure by inhibiting the Nrf2-mediated ferroptosis pathway. Free Radic. Biol. Med..

[35] Du L., Wu Y., Jia Q., Li J., Li Y., Ma H., Fan Z., Guo X., Li L., Peng Y. (2023). Autophagy Suppresses Ferroptosis by Degrading TFR1 to Alleviate Cognitive Dysfunction in Mice with SAE. Cell Mol. Neurobiol..

[36] Fang J., Yuan Q., Du Z., Zhang Q., Yang L., Wang M., Yang W., Yuan C., Yu J., Wu G. (2023). Overexpression of GPX4 attenuates cognitive dysfunction through inhibiting hippocampus ferroptosis and neuroinflammation after traumatic brain injury. Free Radic. Biol. Med..

[37] Weiland A., Wang Y., Wu W., Lan X., Han X., Li Q., Wang J. (2019). Ferroptosis and Its Role in Diverse Brain Diseases. Mol. Neurobiol..

[38] Han T., Xu Y., Sun L., Hashimoto M., Wei J. (2024). Microglial response to aging and neuroinflammation in the development of neurodegenerative diseases. Neural Regen. Res..

[39] Liang Y., Aditi, Onyoni F., Wang H., Gonzales C., Sunyakumthorn P., Wu P., Samir P., Soong L. (2023). Brain transcriptomics reveal the activation of neuroinflammation pathways during acute Orientia tsutsugamushi infection in mice. Front. Immunol..

[40] Merchak A.R., Cahill H.J., Brown L.C., Brown R.M., Rivet-Noor C., Beiter R.M., Slogar E.R., Olgun D.G., Gaultier A. (2023). The activity of the aryl hydrocarbon receptor in T cells tunes the gut microenvironment to sustain autoimmunity and neuroinflammation. PLoS Biol..

[41] Qi X.H., Chen P., Wang Y.J., Zhou Z.P., Liu X.C., Fang H., Wang C.W., Liu J., Liu R.Y., Liu H.K. (2024). Increased cysteinyl-tRNA synthetase drives neuroinflammation in Alzheimer’s disease. Transl. Neurodegener..

[42] Hellani F., Leleu I., Saidi N., Martin N., Lecoeur C., Werkmeister E., Koffi D., Trottein F., Yapo-Ette H., Das B. (2023). Role of astrocyte senescence regulated by the non- canonical autophagy in the neuroinflammation associated to cerebral malaria. Brain Behav. Immun..

[43] Qi R., Wang X. (2020). Inhibition of miR-429 improves neurological recovery of traumatic brain injury mice and attenuates microglial neuroinflammation. Int. Immunopharmacol..

[44] Liu Y., Yang W., Xue J., Chen J., Liu S., Zhang S., Zhang X., Gu X., Dong Y., Qiu P. (2023). Neuroinflammation: The central enabler of postoperative cognitive dysfunction. Biomed. Pharmacother..

[45] Li W., Wu J., Zeng Y., Zheng W. (2023). Neuroinflammation in epileptogenesis: From pathophysiology to therapeutic strategies. Front. Immunol..

[46] Liu P.Y., Li H.Q., Dong M.Q., Gu X.Y., Xu S.Y., Xia S.N., Bao X.Y., Xu Y., Cao X. (2023). Infiltrating myeloid cell-derived properdin markedly promotes microglia-mediated neuroinflammation after ischemic stroke. J. Neuroinflamm..

[47] Rahimian R., Beland L.C., Sato S., Kriz J. (2021). Microglia-derived galectin-3 in neuroinflammation; a bittersweet ligand?. Med. Res. Rev..

[48] Long H.Z., Zhou Z.W., Cheng Y., Luo H.Y., Li F.J., Xu S.G., Gao L.C. (2022). The Role of Microglia in Alzheimer’s Disease From the Perspective of Immune Inflammation and Iron Metabolism. Front. Aging Neurosci..

[49] He L., Zhang R., Yang M., Lu M. (2024). The role of astrocyte in neuroinflammation in traumatic brain injury. Biochim. Biophys. Acta Mol. Basis Dis..

[50] Skok M. (2021). Mesenchymal stem cells as a potential therapeutic tool to cure cognitive impairment caused by neuroinflammation. World J. Stem Cells.

[51] Gonzalez-Fierro C., Fonte C., Dufourd E., Cazaentre V., Aydin S., Engelhardt B., Caspi R.R., Xu B., Martin-Blondel G., Spicer J.A. (2023). Effects of a Small-Molecule Perforin Inhibitor in a Mouse Model of CD8 T Cell-Mediated Neuroinflammation. Neurol. Neuroimmunol. Neuroinflamm..

[52] Puech C., Badran M., Runion A.R., Barrow M.B., Cataldo K., Gozal D. (2023). Cognitive Impairments, Neuroinflammation and Blood-Brain Barrier Permeability in Mice Exposed to Chronic Sleep Fragmentation during the Daylight Period. Int. J. Mol. Sci..

[53] Gharbi T., Zhang Z., Yang G.Y. (2020). The Function of Astrocyte Mediated Extracellular Vesicles in Central Nervous System Diseases. Front. Cell Dev. Biol..

[54] Wang C.S., Kavalali E.T., Monteggia L.M. (2022). BDNF signaling in context: From synaptic regulation to psychiatric disorders. Cell.

[55] Li Y., Pan K., Chen L., Ning J.L., Li X., Yang T., Terrando N., Gu J., Tao G. (2016). Deferoxamine regulates neuroinflammation and iron homeostasis in a mouse model of postoperative cognitive dysfunction. J. Neuroinflamm..

[56] Jung H.Y., Kwon H.J., Kim W., Hwang I.K., Choi G.M., Chang I.B., Kim D.W., Moon S.M. (2021). Tat-Endophilin A1 Fusion Protein Protects Neurons from Ischemic Damage in the Gerbil Hippocampus: A Possible Mechanism of Lipid Peroxidation and Neuroinflammation Mitigation as Well as Synaptic Plasticity. Cells.

[57] He K., Nie L., Ali T., Liu Z., Li W., Gao R., Zhang Z., Liu J., Dai Z., Xie Y. (2023). Adiponectin deficiency accelerates brain aging via mitochondria-associated neuroinflammation. Immun. Ageing.

[58] Li S., Zhou C., Zhu Y., Chao Z., Sheng Z., Zhang Y., Zhao Y. (2021). Ferrostatin-1 alleviates angiotensin II (Ang II)- induced inflammation and ferroptosis in astrocytes. Int. Immunopharmacol..

[59] Li M., Li S.C., Dou B.K., Zou Y.X., Han H.Z., Liu D.X., Ke Z.J., Wang Z.F. (2020). Cycloastragenol upregulates SIRT1 expression, attenuates apoptosis and suppresses neuroinflammation after brain ischemia. Acta Pharmacol. Sin..

[60] Priyanka S.H., Syam Das S., Thushara A.J., Rauf A.A., Indira M. (2018). All Trans Retinoic Acid Attenuates Markers of Neuroinflammation in Rat Brain by Modulation of SIRT1 and NFkappaB. Neurochem. Res..

[61] Song H., Ding Z., Chen J., Chen T., Wang T., Huang J. (2022). The AMPK-SIRT1-FoxO1-NF-kappaB signaling pathway participates in hesperetin-mediated neuroprotective effects against traumatic brain injury via the NLRP3 inflammasome. Immunopharmacol. Immunotoxicol..

[62] Xu J., Zhao L., Zhang X., Ying K., Zhou R., Cai W., Wu X., Jiang H., Xu Q., Miao D. (2023). Salidroside ameliorates acetaminophen-induced acute liver injury through the inhibition of endoplasmic reticulum stress-mediated ferroptosis by activating the AMPK/SIRT1 pathway. Ecotoxicol. Environ. Saf..

[63] Zhang M., Cui J., Chen H., Wang Y., Kuai X., Sun S., Tang Q., Zong F., Chen Q., Wu J. (2023). High-Dosage NMN Promotes Ferroptosis to Suppress Lung Adenocarcinoma Growth through the NAM-Mediated SIRT1-AMPK-ACC Pathway. Cancers.

[64] Siblini Y., Namour F., Oussalah A., Gueant J.L., Chery C. (2022). Stemness of Normal and Cancer Cells: The Influence of Methionine Needs and SIRT1/PGC-1alpha/PPAR-alpha Players. Cells.

[65] Gurd B.J., Menezes E.S., Arhen B.B., Islam H. (2023). Impacts of altered exercise volume, intensity, and duration on the activation of AMPK and CaMKII and increases in PGC-1alpha mRNA. Semin. Cell Dev. Biol..

[66] Liao J., Xie X., Wang N., Wang Y., Zhao J., Chen F., Qu F., Wen W., Miao J., Cui H. (2024). Formononetin promotes fatty acid beta-oxidation to treat non-alcoholic steatohepatitis through SIRT1/PGC-1alpha/PPARalpha pathway. Phytomedicine.

[67] Shi D., Hao Z., Qi W., Jiang F., Liu K., Shi X. (2023). Aerobic exercise combined with chlorogenic acid exerts neuroprotective effects and reverses cognitive decline in Alzheimer’s disease model mice (APP/PS1) via the SIRT1/ /PGC-1alpha/PPARgamma signaling pathway. Front. Aging Neurosci..

[68] Gu L., Ding X., Wang Y., Gu M., Zhang J., Yan S., Li N., Song Z., Yin J., Lu L. (2019). Spexin alleviates insulin resistance and inhibits hepatic gluconeogenesis via the FoxO1/PGC-1alpha pathway in high-fat-diet-induced rats and insulin resistant cells. Int. J. Biol. Sci..

[69] Fan H., Sun Y., Zhang X., Xu Y., Ming Y., Zhang L., Zhao P. (2024). Malvidin promotes PGC-1alpha/Nrf2 signaling to attenuate the inflammatory response and restore mitochondrial activity in septic acute kidney injury. Chem. Biol. Interact..

[70] Zhang X.S., Lu Y., Li W., Tao T., Peng L., Wang W.H., Gao S., Liu C., Zhuang Z., Xia D.Y. (2021). Astaxanthin ameliorates oxidative stress and neuronal apoptosis via SIRT1/NRF2/Prx2/ASK1/p38 after traumatic brain injury in mice. Br. J. Pharmacol..

[71] Yang H., Gu Z.T., Li L., Maegele M., Zhou B.Y., Li F., Zhao M., Zhao K.S. (2017). SIRT1 plays a neuroprotective role in traumatic brain injury in rats via inhibiting the p38 MAPK pathway. Acta Pharmacol. Sin..

[72] Zhao Y., Luo P., Guo Q., Li S., Zhang L., Zhao M., Xu H., Yang Y., Poon W., Fei Z. (2012). Interactions between SIRT1 and MAPK/ERK regulate neuronal apoptosis induced by traumatic brain injury in vitro and in vivo. Exp. Neurol..

[73] Ahmadi A., Ahrari S., Salimian J., Salehi Z., Karimi M., Emamvirdizadeh A., Jamalkandi S.A., Ghanei M. (2023). p38 MAPK signaling in chronic obstructive pulmonary disease pathogenesis and inhibitor therapeutics. Cell Commun. Signal.

[74] Xia M., Zhang Y., Wu H., Zhang Q., Liu Q., Li G., Zhao T., Liu X., Zheng S., Qian Z. (2022). Forsythoside B attenuates neuro-inflammation and neuronal apoptosis by inhibition of NF-kappaB and p38-MAPK signaling pathways through activating Nrf2 post spinal cord injury. Int. Immunopharmacol..

[75] Wang J., Zhang Y. (2018). Neuroprotective effect of berberine agonist against impairment of learning and memory skills in severe traumatic brain injury via Sirt1/p38 MAPK expression. Mol. Med. Rep..

[76] Li C., Wu Z., Xue H., Gao Q., Zhang Y., Wang C., Zhao P. (2022). Ferroptosis contributes to hypoxic-ischemic brain injury in neonatal rats: Role of the SIRT1/Nrf2/GPx4 signaling pathway. CNS Neurosci. Ther..

[77] Chen T., Xu Y.P., Chen Y., Sun S., Yan Z.Z., Wang Y.H. (2023). Arc regulates brain damage and neuroinflammation via Sirt1 signaling following subarachnoid hemorrhage. Brain Res. Bull..

[78] Ruan Q., Wang C., Zhang Y., Sun J. (2023). Brevilin A attenuates cartilage destruction in osteoarthritis mouse model by inhibiting inflammation and ferroptosis via SIRT1/Nrf2/GPX4 signaling pathway. Int. Immunopharmacol..

[79] Zhao L., Jin L., Yang B. (2023). Saikosaponin A alleviates Staphylococcus aureus-induced mastitis in mice by inhibiting ferroptosis via SIRT1/Nrf2 pathway. J. Cell Mol. Med..

[80] Zhao L., Jin L., Yang B. (2023). Diosmetin alleviates S. aureus-induced mastitis by inhibiting SIRT1/GPX4 mediated ferroptosis. Life Sci..

[81] Chen Z., Li J., Peng H., Zhang M., Wu X., Gui F., Li W., Ai F., Yu B., Liu Y. (2023). Meteorin-like/Meteorin-beta protects LPS-induced acute lung injury by activating SIRT1-P53-SLC7A11 mediated ferroptosis pathway. Mol. Med..

[82] Yu M., Li H., Wang B., Wu Z., Wu S., Jiang G., Wang H., Huang Y. (2023). Baicalein ameliorates polymyxin B-induced acute renal injury by inhibiting ferroptosis via regulation of SIRT1/p53 acetylation. Chem. Biol. Interact..

[83] Liu Q., Liu Y., Li Y., Hong Z., Li S., Liu C. (2023). PUM2 aggravates the neuroinflammation and brain damage induced by ischemia-reperfusion through the SLC7A11-dependent inhibition of ferroptosis via suppressing the SIRT1. Mol. Cell Biochem..

[84] Engeland K. (2022). Cell cycle regulation: p53-p21-RB signaling. Cell Death Differ..

[85] Rodencal J., Kim N., He A., Li V.L., Lange M., He J., Tarangelo A., Schafer Z.T., Olzmann J.A., Long J.Z. (2024). Sensitization of cancer cells to ferroptosis coincident with cell cycle arrest. Cell Chem. Biol..

[86] Yan C., Yang S., Shao S., Zu R., Lu H., Chen Y., Zhou Y., Ying X., Xiang S., Zhang P. (2024). Exploring the anti-ferroptosis mechanism of Kai-Xin-San against Alzheimer’s disease through integrating network pharmacology, bioinformatics, and experimental validation strategy in vivo and in vitro. J. Ethnopharmacol..

[87] Yuan B., Zhao X.D., Shen J.D., Chen S.J., Huang H.Y., Zhou X.M., Han Y.L., Zhou L.J., Lu X.J., Wu Q. (2022). Activation of SIRT1 Alleviates Ferroptosis in the Early Brain Injury after Subarachnoid Hemorrhage. Oxidative Med. Cell. Longev..

[88] Zhang J., Zhu Q., Peng Z., Li X.J., Ding P.F., Gao S., Sheng B., Liu Y., Lu Y., Zhuang Z. (2024). Menaquinone-4 attenuates ferroptosis by upregulating DHODH through activation of SIRT1 after subarachnoid hemorrhage. Free Radic. Biol. Med..

[89] Peng X., Wang J., Peng J., Jiang H., Le K. (2022). Resveratrol Improves Synaptic Plasticity in Hypoxic-Ischemic Brain Injury in Neonatal Mice via Alleviating SIRT1/NF-kappaB Signaling-Mediated Neuroinflammation. J. Mol. Neurosci..

[90] Chen X., Chen C., Fan S., Wu S., Yang F., Fang Z., Fu H., Li Y. (2018). Omega-3 polyunsaturated fatty acid attenuates the inflammatory response by modulating microglia polarization through SIRT1-mediated deacetylation of the HMGB1/NF-kappaB pathway following experimental traumatic brain injury. J. Neuroinflamm..

[91] Chen Y., Peng F., Yang C., Hou H., Xing Z., Chen J., Liu L., Peng C., Li D. (2023). SIRT1 activation by 2,3,5,6-tetramethylpyrazine alleviates neuroinflammation via inhibiting M1 microglia polarization. Front. Immunol..

[92] Zhao Y.S., Li J.Y., Li Z.C., Wang L.L., Gan C.L., Chen J., Jiang S.Y., Aschner M., Ou S.Y., Jiang Y.M. (2023). Sodium Para-aminosalicylic Acid Inhibits Lead-Induced Neuroinflammation in Brain Cortex of Rats by Modulating SIRT1/HMGB1/NF-kappaB Pathway. Neurochem. Res..

[93] Zhang W., Qian S., Tang B., Kang P., Zhang H., Shi C. (2023). Resveratrol inhibits ferroptosis and decelerates heart failure progression via Sirt1/p53 pathway activation. J. Cell Mol. Med..

[94] Zou P., Liu X., Li G., Wang Y. (2018). Resveratrol pretreatment attenuates traumatic brain injury in rats by suppressing NLRP3 inflammasome activation via SIRT1. Mol. Med. Rep..

[95] Grabowska A.D., Watroba M., Witkowska J., Mikulska A., Sepulveda N., Szukiewicz D. (2023). Interplay between Systemic Glycemia and Neuroprotective Activity of Resveratrol in Modulating Astrocyte SIRT1 Response to Neuroinflammation. Int. J. Mol. Sci..

[96] Yan R., Lin B., Jin W., Tang L., Hu S., Cai R. (2023). NRF2, a Superstar of Ferroptosis. Antioxidants.

[97] Martin D., Rojo A.I., Salinas M., Diaz R., Gallardo G., Alam J., De Galarreta C.M., Cuadrado A. (2004). Regulation of heme oxygenase-1 expression through the phosphatidylinositol 3-kinase/Akt pathway and the Nrf2 transcription factor in response to the antioxidant phytochemical carnosol. J. Biol. Chem..

[98] Xie X., Wang F., Ge W., Meng X., Fan L., Zhang W., Wang Z., Ding M., Gu S., Xing X. (2023). Scutellarin attenuates oxidative stress and neuroinflammation in cerebral ischemia/reperfusion injury through PI3K/Akt-mediated Nrf2 signaling pathways. Eur. J. Pharmacol..

[99] Cheng Y., Gao Y., Li J., Rui T., Li Q., Chen H., Jia B., Song Y., Gu Z., Wang T. (2023). TrkB agonist N-acetyl serotonin promotes functional recovery after traumatic brain injury by suppressing ferroptosis via the PI3K/Akt/Nrf2/Ferritin H pathway. Free Radic. Biol. Med..

[100] Wu T., Zhao F., Gao B., Tan C., Yagishita N., Nakajima T., Wong P.K., Chapman E., Fang D., Zhang D.D. (2014). Hrd1 suppresses Nrf2-mediated cellular protection during liver cirrhosis. Genes. Dev..

[101] Feng X., Ma W., Zhu J., Jiao W., Wang Y. (2021). Dexmedetomidine alleviates early brain injury following traumatic brain injury by inhibiting autophagy and neuroinflammation through the ROS/Nrf2 signaling pathway. Mol. Med. Rep..

[102] Wang C., Chen S., Guo H., Jiang H., Liu H., Fu H., Wang D. (2022). Forsythoside A Mitigates Alzheimer’s-like Pathology by Inhibiting Ferroptosis-mediated Neuroinflammation via Nrf2/GPX4 Axis Activation. Int. J. Biol. Sci..

[103] Lee J., Hyun D.H. (2023). The Interplay between Intracellular Iron Homeostasis and Neuroinflammation in Neurodegenerative Diseases. Antioxidants.

[104] Cheng H., Wang P., Wang N., Dong W., Chen Z., Wu M., Wang Z., Yu Z., Guan D., Wang L. (2023). Neuroprotection of NRF2 against Ferroptosis after Traumatic Brain Injury in Mice. Antioxidants.

[105] Pietsch E.C., Chan J.Y., Torti F.M., Torti S.V. (2003). Nrf2 mediates the induction of ferritin H in response to xenobiotics and cancer chemopreventive dithiolethiones. J. Biol. Chem..

[106] Zhang Y., Lan J., Zhao D., Ruan C., Zhou J., Tan H., Bao Y. (2023). Netrin-1 upregulates GPX4 and prevents ferroptosis after traumatic brain injury via the UNC5B/Nrf2 signaling pathway. CNS Neurosci. Ther..

[107] Ma X.Z., Chen L.L., Qu L., Li H., Wang J., Song N., Xie J.X. (2024). Gut microbiota-induced CXCL1 elevation triggers early neuroinflammation in the substantia nigra of Parkinsonian mice. Acta Pharmacol. Sin..

[108] Duan C., Jiao D., Wang H., Wu Q., Men W., Yan H., Li C. (2022). Activation of the PPARgamma Prevents Ferroptosis-Induced Neuronal Loss in Response to Intracerebral Hemorrhage Through Synergistic Actions With the Nrf2. Front. Pharmacol..

[109] Lv J., Xu S., Meng C., Wang Y., Ji L., Li X., Wang X., Li Q. (2023). Ferroptosis participated in hippocampal neuroinflammation damage of in offspring rats after maternal sleep deprivation. J. Neuroimmunol..

[110] Cui Y., Zhang Z., Zhou X., Zhao Z., Zhao R., Xu X., Kong X., Ren J., Yao X., Wen Q. (2021). Microglia and macrophage exhibit attenuated inflammatory response and ferroptosis resistance after RSL3 stimulation via increasing Nrf2 expression. J. Neuroinflammation.

[111] Gao Q., Zhao Y., Luo R., Su M., Zhang C., Li C., Liu B., Zhou X. (2023). Intrathecal umbilical cord mesenchymal stem cells injection alleviates neuroinflammation and oxidative stress in the cyclophosphamide-induced interstitial cystitis rats through the Sirt1/Nrf2/HO-1 pathway. Life Sci..

[112] Zhao X.J., Zhu H.Y., Wang X.L., Lu X.W., Pan C.L., Xu L., Liu X., Xu N., Zhang Z.Y. (2022). Oridonin Ameliorates Traumatic Brain Injury-Induced Neurological Damage by Improving Mitochondrial Function and Antioxidant Capacity and Suppressing Neuroinflammation through the Nrf2 Pathway. J. Neurotrauma.

[113] Wang L., Ding Y.Y., Wu Y.Q., Zhao C., Wu J., Wang W.J., Meng F.H. (2023). Koumine ameliorates neuroinflammation by regulating microglia polarization via activation of Nrf2/HO-1 pathway. Biomed. Pharmacother..

[114] Guo Y., Ou C., Zhang N., Liu Q., Xiong K., Yu J., Cheng H., Chen L., Ma M., Xu J. (2023). Roflumilast attenuates neuroinflammation post retinal ischemia/reperfusion injury by regulating microglia phenotype via the Nrf2/STING/NF-kappaB pathway. Int. Immunopharmacol..

[115] Xiaodan S., Peiyan Z., Hui L., Yan L., Ying C. (2023). NRF2 participates in the suppressive tumor immune microenvironment of KRAS/KEAP1 co-mutant non-small cell lung cancer by inhibiting the STING pathway. Genes. Dis..

[116] Yang T., Qu X., Zhao J., Wang X., Wang Q., Dai J., Zhu C., Li J., Jiang L. (2023). Macrophage PTEN controls STING-induced inflammation and necroptosis through NICD/NRF2 signaling in APAP-induced liver injury. Cell Commun. Signal..

[117] He L., Liu D., Zhou W., Han Y., Ju Y., Liu H., Chen Y., Yu J., Wang L., Wang J. (2023). The innate immune sensor STING accelerates neointima formation via NF-kappaB signaling pathway. Int. Immunopharmacol..

[118] Bellezza I., Tucci A., Galli F., Grottelli S., Mierla A.L., Pilolli F., Minelli A. (2012). Inhibition of NF-kappaB nuclear translocation via HO-1 activation underlies alpha-tocopheryl succinate toxicity. J. Nutr. Biochem..

[119] Gao Y., Zhang H., Wang J., Li F., Li X., Li T., Wang C., Li L., Peng R., Liu L. (2023). Annexin A5 ameliorates traumatic brain injury-induced neuroinflammation and neuronal ferroptosis by modulating the NF-kB/HMGB1 and Nrf2/HO-1 pathways. Int. Immunopharmacol..

[120] Capece D., Verzella D., Flati I., Arboretto P., Cornice J., Franzoso G. (2022). NF-kappaB: Blending metabolism, immunity, and inflammation. Trends Immunol..

[121] Singh S.S., Rai S.N., Birla H., Zahra W., Rathore A.S., Singh S.P. (2020). NF-kappaB-Mediated Neuroinflammation in Parkinson’s Disease and Potential Therapeutic Effect of Polyphenols. Neurotox. Res..

[122] Ji H.Z., Chen L., Ren M., Li S., Liu T.Y., Chen H.J., Yu H.H., Sun Y. (2023). CXCL8 Promotes Endothelial-to-Mesenchymal Transition of Endothelial Cells and Protects Cells from Erastin-Induced Ferroptosis via CXCR2-Mediated Activation of the NF-kappaB Signaling Pathway. Pharmaceuticals.

[123] Tamiya H., Urushihara N., Shizuma K., Ogawa H., Nakai S., Wakamatsu T., Takenaka S., Kakunaga S. (2023). SHARPIN Enhances Ferroptosis in Synovial Sarcoma Cells via NF-kappaB- and PRMT5-Mediated PGC1alpha Reduction. Cancers.

[124] Kaur U., Banerjee P., Bir A., Sinha M., Biswas A., Chakrabarti S. (2015). Reactive oxygen species, redox signaling and neuroinflammation in Alzheimer’s disease: The NF-kappaB connection. Curr. Top. Med. Chem..

[125] Chen P.H., Wu J., Ding C.C., Lin C.C., Pan S., Bossa N., Xu Y., Yang W.H., Mathey-Prevot B., Chi J.T. (2020). Kinome screen of ferroptosis reveals a novel role of ATM in regulating iron metabolism. Cell Death Differ..

[126] Jia H., Liu X., Cao Y., Niu H., Lan Z., Li R., Li F., Sun D., Shi M., Wa L. (2023). Deferoxamine ameliorates neurological dysfunction by inhibiting ferroptosis and neuroinflammation after traumatic brain injury. Brain Res..

[127] Hambright W.S., Fonseca R.S., Chen L., Na R., Ran Q. (2017). Ablation of ferroptosis regulator glutathione peroxidase 4 in forebrain neurons promotes cognitive impairment and neurodegeneration. Redox Biol..

[128] Ren Y., Zhu D., Han X., Zhang Q., Chen B., Zhou P., Wei Z., Zhang Z., Cao Y., Zou H. (2023). HMGB1: A double-edged sword and therapeutic target in the female reproductive system. Front. Immunol..

[129] Chen Y., He W., Wei H., Chang C., Yang L., Meng J., Long T., Xu Q., Zhang C. (2023). Srs11-92, a ferrostatin-1 analog, improves oxidative stress and neuroinflammation via Nrf2 signal following cerebral ischemia/reperfusion injury. CNS Neurosci. Ther..

[130] Lee S., Ju I.G., Choi Y., Park S., Oh M.S. (2021). Trichosanthis Semen Suppresses Lipopolysaccharide-Induced Neuroinflammation by Regulating the NF-kappaB Signaling Pathway and HO-1 Expression in Microglia. Toxins.

[131] Zhang S.S., Liu M., Liu D.N., Shang Y.F., Wang Y.H., Du G.H. (2022). ST2825, a Small Molecule Inhibitor of MyD88, Suppresses NF-kappaB Activation and the ROS/NLRP3/Cleaved Caspase-1 Signaling Pathway to Attenuate Lipopolysaccharide-Stimulated Neuroinflammation. Molecules.

[132] Guan F., Zhou X., Li P., Wang Y., Liu M., Li F., Cui Y., Huang T., Yao M., Zhang Y. (2019). MG53 attenuates lipopolysaccharide-induced neurotoxicity and neuroinflammation via inhibiting TLR4/NF-kappaB pathway in vitro and in vivo. Prog. Neuropsychopharmacol. Biol. Psychiatry.

[133] An Q., Xia J., Pu F., Shi S. (2024). MCPIP1 alleviates depressive-like behaviors in mice by inhibiting the TLR4/TRAF6/NF-kappaB pathway to suppress neuroinflammation. Mol. Med. Rep..

[134] Xu Y., Zhang J., Gao F., Cheng W., Zhang Y., Wei C., Zhang S., Gao X. (2023). Engeletin alleviates cerebral ischemia reperfusion-induced neuroinflammation via the HMGB1/TLR4/NF-kappaB network. J. Cell Mol. Med..

[135] Zhang Y., Ye P., Zhu H., Gu L., Li Y., Feng S., Zeng Z., Chen Q., Zhou B., Xiong X. (2023). Neutral polysaccharide from Gastrodia elata alleviates cerebral ischemia-reperfusion injury by inhibiting ferroptosis-mediated neuroinflammation via the NRF2/HO-1 signaling pathway. CNS Neurosci. Ther..

[136] Wang M., Tang G., Zhou C., Guo H., Hu Z., Hu Q., Li G. (2023). Revisiting the intersection of microglial activation and neuroinflammation in Alzheimer’s disease from the perspective of ferroptosis. Chem. Biol. Interact..

[137] Pozniak P.D., White M.K., Khalili K. (2014). TNF-alpha/NF-kappaB signaling in the CNS: Possible connection to EPHB2. J. Neuroimmune Pharmacol..

[138] Wang X., Li S., Yu J., Wang W., Du Z., Gao S., Ma Y., Tang R., Liu T., Ma S. (2023). Saikosaponin B2 ameliorates depression-induced microglia activation by inhibiting ferroptosis-mediated neuroinflammation and ER stress. J. Ethnopharmacol..

[139] Abdulaal W.H., Omar U.M., Zeyadi M., El-Agamy D.S., Alhakamy N.A., Ibrahim S.R.M., Almalki N.A.R., Asfour H.Z., Al-Rabia M.W., Mohamed G.A. (2024). Modulation of the crosstalk between Keap1/Nrf2/HO-1 and NF-kappaB signaling pathways by Tomatidine protects against inflammation/oxidative stress-driven fulminant hepatic failure in mice. Int. Immunopharmacol..

[140] Xiao X., Jiang Y., Liang W., Wang Y., Cao S., Yan H., Gao L., Zhang L. (2019). miR-212-5p attenuates ferroptotic neuronal death after traumatic brain injury by targeting Ptgs2. Mol. Brain.

[141] Zhang X.H., Cui H., Zheng S.M., Lu Y., Yuan H.W., Zhang L., Wang H.H., Du R.S. (2023). Electroacupuncture regulates microglial polarization via inhibiting NF-kappaB/COX2 pathway following traumatic brain injury. Brain Res..

[142] Prabhakaran J., Molotkov A., Mintz A., Mann J.J. (2021). Progress in PET Imaging of Neuroinflammation Targeting COX-2 Enzyme. Molecules.

[143] Hakan T., Toklu H.Z., Biber N., Ozevren H., Solakoglu S., Demirturk P., Aker F.V. (2010). Effect of COX-2 inhibitor meloxicam against traumatic brain injury-induced biochemical, histopathological changes and blood-brain barrier permeability. Neurol. Res..

[144] Liu J., Zhou Y., Xie C., Li C., Ma L., Zhang Y. (2023). Anti-Ferroptotic Effects of bone Marrow Mesenchymal Stem Cell-Derived Extracellular Vesicles Loaded with Ferrostatin-1 in Cerebral ischemia-reperfusion Injury Associate with the GPX4/COX-2 Axis. Neurochem. Res..

[145] Moussa N., Dayoub N. (2023). Exploring the role of COX-2 in Alzheimer’s disease: Potential therapeutic implications of COX-2 inhibitors. Saudi Pharm. J..

[146] Niranjan R., Mishra K.P., Thakur A.K. (2018). Inhibition of Cyclooxygenase-2 (COX-2) Initiates Autophagy and Potentiates MPTP-Induced Autophagic Cell Death of Human Neuroblastoma Cells, SH-SY5Y: An Inside in the Pathology of Parkinson’s Disease. Mol. Neurobiol..

[147] Ayala A., Munoz M.F., Arguelles S. (2014). Lipid peroxidation: Production, metabolism, and signaling mechanisms of malondialdehyde and 4-hydroxy-2-nonenal. Oxidative Med. Cell. Longev..

[148] Song H., Park J., Bui P.T.C., Choi K., Gye M.C., Hong Y.C., Kim J.H., Lee Y.J. (2017). Bisphenol A induces COX-2 through the mitogen-activated protein kinase pathway and is associated with levels of inflammation-related markers in elderly populations. Environ. Res..

[149] Cherukuri D.P., Goulet A.C., Inoue H., Nelson M.A. (2005). Selenomethionine regulates cyclooxygenase-2 (COX-2) expression through nuclear factor-kappa B (NF-kappaB) in colon cancer cells. Cancer Biol. Ther..

[150] Chen Y.F., Wang Y.W., Huang W.S., Lee M.M., Wood W.G., Leung Y.M., Tsai H.Y. (2016). Trans-Cinnamaldehyde, An Essential Oil in Cinnamon Powder, Ameliorates Cerebral Ischemia-Induced Brain Injury via Inhibition of Neuroinflammation Through Attenuation of iNOS, COX-2 Expression and NFkappa-B Signaling Pathway. Neuromol. Med..

[151] Yan J.J., Du G.H., Qin X.M., Gao L. (2020). Baicalein attenuates the neuroinflammation in LPS-activated BV-2 microglial cells through suppression of pro-inflammatory cytokines, COX2/NF-kappaB expressions and regulation of metabolic abnormality. Int. Immunopharmacol..

[152] Haile M., Boutajangout A., Chung K., Chan J., Stolper T., Vincent N., Batchan M., D’Urso J., Lin Y., Kline R. (2016). The Cox-2 Inhibitor Meloxicam Ameliorates Neuroinflammation and Depressive Behavior in Adult Mice after Splenectomy. J. Neurophysiol. Neurol. Disord..

[153] Zhou Y., Zhou H., Hua L., Hou C., Jia Q., Chen J., Zhang S., Wang Y., He S., Jia E. (2021). Verification of ferroptosis and pyroptosis and identification of PTGS2 as the hub gene in human coronary artery atherosclerosis. Free Radic. Biol. Med..

[154] Cao Y., Li Y., He C., Yan F., Li J.R., Xu H.Z., Zhuang J.F., Zhou H., Peng Y.C., Fu X.J. (2021). Selective Ferroptosis Inhibitor Liproxstatin-1 Attenuates Neurological Deficits and Neuroinflammation After Subarachnoid Hemorrhage. Neurosci. Bull..

[155] Zou C., Xu F., Shen J., Xu S. (2022). Identification of a Ferroptosis-Related Prognostic Gene PTGS2 Based on Risk Modeling and Immune Microenvironment of Early-Stage Cervical Cancer. J. Oncol..

[156] Zhang T., Yang M., Ma C., Wei X., Zhang Z. (2023). BACH1 encourages ferroptosis by activating KDM4C-mediated COX2 demethylation after cerebral ischemia-reperfusion injury. Eur. J. Neurosci..

[157] Sun Y., Chen P., Zhai B., Zhang M., Xiang Y., Fang J., Xu S., Gao Y., Chen X., Sui X. (2020). The emerging role of ferroptosis in inflammation. Biomed. Pharmacother..

[158] Liu T., Shu J., Liu Y., Xie J., Li T., Li H., Li L. (2022). Atorvastatin attenuates ferroptosis-dependent myocardial injury and inflammation following coronary microembolization via the Hif1a/Ptgs2 pathway. Front. Pharmacol..

[159] Gao F., Zhao Y., Zhang B., Xiao C., Sun Z., Gao Y., Dou X. (2022). Suppression of lncRNA Gm47283 attenuates myocardial infarction via miR-706/ Ptgs2/ferroptosis axis. Bioengineered.

[160] Liu C., Lu J., Yuan T., Xie L., Zhang L. (2023). EPC-exosomal miR-26a-5p improves airway remodeling in COPD by inhibiting ferroptosis of bronchial epithelial cells via PTGS2/PGE2 signaling pathway. Sci. Rep..

[161] Xue Z., Zhang Z., Liu H., Li W., Guo X., Zhang Z., Liu Y., Jia L., Li Y., Ren Y. (2019). lincRNA-Cox2 regulates NLRP3 inflammasome and autophagy mediated neuroinflammation. Cell Death Differ..

[162] Jiang H., Sun Z., Zhu X., Li F., Chen Q. (2023). Essential genes Ptgs2, Tlr4, and Ccr2 regulate neuro-inflammation during the acute phase of cerebral ischemic in mice. Sci. Rep..

[163] Ma P., Li Y., Zhang W., Fang F., Sun J., Liu M., Li K., Dong L. (2019). Long Non-coding RNA MALAT1 Inhibits Neuron Apoptosis and Neuroinflammation While Stimulates Neurite Outgrowth and Its Correlation With MiR-125b Mediates PTGS2, CDK5 and FOXQ1 in Alzheimer’s Disease. Curr. Alzheimer Res..

[164] Yang H., Wang H., Shu Y., Li X. (2018). miR-103 Promotes Neurite Outgrowth and Suppresses Cells Apoptosis by Targeting Prostaglandin-Endoperoxide Synthase 2 in Cellular Models of Alzheimer’s Disease. Front. Cell Neurosci..

[165] Li Y., Wang J., Chen S., Wu P., Xu S., Wang C., Shi H., Bihl J. (2020). miR-137 boosts the neuroprotective effect of endothelial progenitor cell-derived exosomes in oxyhemoglobin-treated SH-SY5Y cells partially via COX2/PGE2 pathway. Stem Cell Res. Ther..

[166] Yi T.T., Zhang L.M., Huang X.N. (2023). Glycyrrhizic acid protects against temporal lobe epilepsy in young rats by regulating neuronal ferroptosis through the miR-194-5p/PTGS2 axis. Kaohsiung J. Med. Sci..

[167] Kim M.E., Jung I., Na J.Y., Lee Y., Lee J., Lee J.S., Lee J.S. (2018). Pseudane-VII Regulates LPS-Induced Neuroinflammation in Brain Microglia Cells through the Inhibition of iNOS Expression. Molecules.

[168] Hu C., He M., Chen M., Xu Q., Li S., Cui Y., Qiu X., Tian W. (2022). Amelioration of Neuropathic Pain and Attenuation of Neuroinflammation Responses by Tetrahydropalmatine Through the p38MAPK/NF-kappaB/iNOS Signaling Pathways in Animal and Cellular Models. Inflammation.

[169] Wang S.S., Jia J., Wang Z. (2018). Mesenchymal Stem Cell-Derived Extracellular Vesicles Suppresses iNOS Expression and Ameliorates Neural Impairment in Alzheimer’s Disease Mice. J. Alzheimers Dis..

[170] Dong Y., Han L.L., Xu Z.X. (2018). Suppressed microRNA-96 inhibits iNOS expression and dopaminergic neuron apoptosis through inactivating the MAPK signaling pathway by targeting CACNG5 in mice with Parkinson’s disease. Mol. Med..

[171] Zhou M., Wang C.M., Yang W.L., Wang P. (2013). Microglial CD14 activated by iNOS contributes to neuroinflammation in cerebral ischemia. Brain Res..

[172] Zheng S., Wang C., Lin L., Mu S., Liu H., Hu X., Chen X., Wang S. (2023). TNF-alpha Impairs Pericyte-Mediated Cerebral Microcirculation via the NF-kappaB/iNOS Axis after Experimental Traumatic Brain Injury. J. Neurotrauma.

[173] Adeoluwa O.A., Olayinka J.N., Adeoluwa G.O., Akinluyi E.T., Adeniyi F.R., Fafure A., Nebo K., Edem E.E., Eduviere A.T., Abubakar B. (2023). Quercetin abrogates lipopolysaccharide-induced depressive-like symptoms by inhibiting neuroinflammation via microglial NLRP3/NFkappaB/iNOS signaling pathway. Behav. Brain Res..

[174] Gong L., Zhu T., Chen C., Xia N., Yao Y., Ding J., Xu P., Li S., Sun Z., Dong X. (2022). Miconazole exerts disease-modifying effects during epilepsy by suppressing neuroinflammation via NF-kappaB pathway and iNOS production. Neurobiol. Dis..

[175] Urrutia P.J., Borquez D.A., Nunez M.T. (2021). Inflaming the Brain with Iron. Antioxidants.

[176] Ko C.J., Gao S.L., Lin T.K., Chu P.Y., Lin H.Y. (2021). Ferroptosis as a Major Factor and Therapeutic Target for Neuroinflammation in Parkinson’s Disease. Biomedicines.

[177] Qu W., Cheng Y., Peng W., Wu Y., Rui T., Luo C., Zhang J. (2022). Targeting iNOS Alleviates Early Brain Injury After Experimental Subarachnoid Hemorrhage via Promoting Ferroptosis of M1 Microglia and Reducing Neuroinflammation. Mol. Neurobiol..

[178] Dar H.H., Anthonymuthu T.S., Ponomareva L.A., Souryavong A.B., Shurin G.V., Kapralov A.O., Tyurin V.A., Lee J.S., Mallampalli R.K., Wenzel S.E. (2021). A new thiol-independent mechanism of epithelial host defense against Pseudomonas aeruginosa: iNOS/NO(*) sabotage of theft-ferroptosis. Redox Biol..

[179] Mikulska-Ruminska K., Anthonymuthu T.S., Levkina A., Shrivastava I.H., Kapralov A.A., Bayir H., Kagan V.E., Bahar I. (2021). NO(●) Represses the Oxygenation of Arachidonoyl PE by 15LOX/PEBP1: Mechanism and Role in Ferroptosis. Int. J. Mol. Sci..

[180] Zi Y., Wang X., Zi Y., Yu H., Lan Y., Fan Y., Ren C., Liao K., Chen H. (2023). Cigarette smoke induces the ROS accumulation and iNOS activation through deactivation of Nrf-2/SIRT3 axis to mediate the human bronchial epithelium ferroptosis. Free Radic. Biol. Med..

[181] Wen Y., Chen H., Zhang L., Wu M., Zhang F., Yang D., Shen J., Chen J. (2021). Glycyrrhetinic acid induces oxidative/nitrative stress and drives ferroptosis through activating NADPH oxidases and iNOS, and depriving glutathione in triple-negative breast cancer cells. Free Radic. Biol. Med..

[182] Kapralov A.A., Yang Q., Dar H.H., Tyurina Y.Y., Anthonymuthu T.S., Kim R., St Croix C.M., Mikulska-Ruminska K., Liu B., Shrivastava I.H. (2020). Redox lipid reprogramming commands susceptibility of macrophages and microglia to ferroptotic death. Nat. Chem. Biol..

[183] Bayir H., Kagan V.E., Borisenko G.G., Tyurina Y.Y., Janesko K.L., Vagni V.A., Billiar T.R., Williams D.L., Kochanek P.M. (2005). Enhanced oxidative stress in iNOS-deficient mice after traumatic brain injury: Support for a neuroprotective role of iNOS. J. Cereb. Blood Flow. Metab..

[184] Chen X., Pang X., Yeo A.J., Xie S., Xiang M., Shi B., Yu G., Li C. (2022). The Molecular Mechanisms of Ferroptosis and Its Role in Blood-Brain Barrier Dysfunction. Front. Cell Neurosci..

[185] Tian D., Li W., Heffron C.L., Wang B., Mahsoub H.M., Sooryanarain H., Hassebroek A.M., Clark-Deener S., LeRoith T., Meng X.J. (2022). Hepatitis E virus infects brain microvascular endothelial cells, crosses the blood-brain barrier, and invades the central nervous system. Pro. Natl. Acad. Sci. USA.

[186] Huang Y.-T., Hsu Y.-T., Wu P.-Y., Yeh Y.-M., Lin P.-C., Hsu K.-F., Shen M.-R. (2024). Tight junction protein cingulin variant is associated with cancer susceptibility by overexpressed IQGAP1 and Rac1-dependent epithelial-mesenchymal transition. J. Exp. Clin. Cancer Res..

[187] Wang P., Jin L., Zhang M., Wu Y., Duan Z., Guo Y., Wang C., Guo Y., Chen W., Liao Z. (2023). Blood–brain barrier injury and neuroinflammation induced by SARS-CoV-2 in a lung–brain microphysiological system. Nat. Biomed. Eng..

[188] Li W., Tiedt S., Lawrence J.H., Harrington M.E., Musiek E.S., Lo E.H. (2024). Circadian Biology and the Neurovascular Unit. Circ. Res..

[189] Zhao Y., Liu Y., Xu Y., Li K., Zhou L., Qiao H., Xu Q., Zhao J. (2023). The Role of Ferroptosis in Blood-Brain Barrier Injury. Cell Mol. Neurobiol..

[190] Wang P., Ren Q., Shi M., Liu Y., Bai H., Chang Y.Z. (2022). Overexpression of Mitochondrial Ferritin Enhances Blood-Brain Barrier Integrity Following Ischemic Stroke in Mice by Maintaining Iron Homeostasis in Endothelial Cells. Antioxidants.

[191] You L., Yu P.P., Dong T., Guo W., Chang S., Zheng B., Ci Y., Wang F., Yu P., Gao G. (2022). Astrocyte-derived hepcidin controls iron traffic at the blood-brain-barrier via regulating ferroportin 1 of microvascular endothelial cells. Cell Death Dis..

[192] Rand D., Ravid O., Atrakchi D., Israelov H., Bresler Y., Shemesh C., Omesi L., Liraz-Zaltsman S., Gosselet F., Maskrey T.S. (2021). Endothelial Iron Homeostasis Regulates Blood-Brain Barrier Integrity via the HIF2alpha-Ve-Cadherin Pathway. Pharmaceutics.

[193] Li J., Li M., Ge Y., Chen J., Ma J., Wang C., Sun M., Wang L., Yao S., Yao C. (2022). beta-amyloid protein induces mitophagy-dependent ferroptosis through the CD36/PINK/PARKIN pathway leading to blood-brain barrier destruction in Alzheimer’s disease. Cell Biosci..

[194] Liu Q., Song T., Chen B., Zhang J., Li W. (2023). Ferroptosis of brain microvascular endothelial cells contributes to hypoxia-induced blood-brain barrier injury. FASEB J..

[195] Xu S., Li X., Li Y., Li X., Lv E., Zhang X., Shi Y., Wang Y. (2023). Neuroprotective effect of Dl-3-n-butylphthalide against ischemia-reperfusion injury is mediated by ferroptosis regulation via the SLC7A11/GSH/GPX4 pathway and the attenuation of blood-brain barrier disruption. Front. Aging Neurosci..

[196] Fang J., Yuan Q., Du Z., Fei M., Zhang Q., Yang L., Wang M., Yang W., Yu J., Wu G. (2022). Ferroptosis in brain microvascular endothelial cells mediates blood-brain barrier disruption after traumatic brain injury. Biochem. Biophys. Res. Commun..

[197] Meng S., Cao H., Huang Y., Shi Z., Li J., Wang Y., Zhang Y., Chen S., Shi H., Gao Y. (2023). ASK1-K716R reduces neuroinflammation and white matter injury via preserving blood-brain barrier integrity after traumatic brain injury. J. Neuroinflamm..

[198] Villalba N., Ma Y., Gahan S.A., Joly-Amado A., Spence S., Yang X., Nash K.R., Yuan S.Y. (2023). Lung infection by Pseudomonas aeruginosa induces neuroinflammation and blood-brain barrier dysfunction in mice. J. Neuroinflamm..

[199] Liao Y.C., Wang J.W., Guo C., Bai M., Ran Z., Wen L.M., Ju B.W., Ding Y., Hu J.P., Yang J.H. (2023). Cistanche tubulosa alleviates ischemic stroke-induced blood-brain barrier damage by modulating microglia-mediated neuroinflammation. J. Ethnopharmacol..

[200] Erickson M.A., Shulyatnikova T., Banks W.A., Hayden M.R. (2023). Ultrastructural Remodeling of the Blood-Brain Barrier and Neurovascular Unit by Lipopolysaccharide-Induced Neuroinflammation. Int. J. Mol. Sci..

[201] Larochelle J., Yang C., Liu L., Candelario-Jalil E. (2022). An Unexplored Role for MMP-7 (Matrix Metalloproteinase-7) in Promoting Gut Permeability After Ischemic Stroke. Stroke.

[202] Liu Y., Mu Y., Li Z., Yong V.W., Xue M. (2022). Extracellular matrix metalloproteinase inducer in brain ischemia and intracerebral hemorrhage. Front. Immunol..

[203] Isik S., Yeman Kiyak B., Akbayir R., Seyhali R., Arpaci T. (2023). Microglia Mediated Neuroinflammation in Parkinson’s Disease. Cells.

[204] Yu H., Chang Q., Sun T., He X., Wen L., An J., Feng J., Zhao Y. (2023). Metabolic reprogramming and polarization of microglia in Parkinson’s disease: Role of inflammasome and iron. Ageing Res. Rev..

[205] Liu S., Gao X., Zhou S. (2022). New Target for Prevention and Treatment of Neuroinflammation: Microglia Iron Accumulation and Ferroptosis. ASN Neuro.

[206] Zhou X., Zhao R., Lv M., Xu X., Liu W., Li X., Gao Y., Zhao Z., Zhang Z., Li Y. (2023). ACSL4 promotes microglia-mediated neuroinflammation by regulating lipid metabolism and VGLL4 expression. Brain Behav. Immun..

[207] Wu Y., Eisel U.L.M. (2023). Microglia-Astrocyte Communication in Alzheimer’s Disease. J. Alzheimers Dis..

[208] Manu D.R., Slevin M., Barcutean L., Forro T., Boghitoiu T., Balasa R. (2023). Astrocyte Involvement in Blood-Brain Barrier Function: A Critical Update Highlighting Novel, Complex, Neurovascular Interactions. Int. J. Mol. Sci..

[209] Juric D.M., Krzan M., Lipnik-Stangelj M. (2016). Histamine and astrocyte function. Pharmacol. Res..

[210] Blackburn D., Sargsyan S., Monk P.N., Shaw P.J. (2009). Astrocyte function and role in motor neuron disease: A future therapeutic target?. Glia.

[211] Murphy-Royal C., Ching S., Papouin T. (2023). A conceptual framework for astrocyte function. Nat. Neurosci..

[212] Verkhratsky A., Butt A., Li B., Illes P., Zorec R., Semyanov A., Tang Y., Sofroniew M.V. (2023). Astrocytes in human central nervous system diseases: A frontier for new therapies. Signal Transduct. Target. Ther..

[213] Wang N., Zhao Y., Wu M., Li N., Yan C., Guo H., Li Q., Li Q., Wang Q. (2024). Gemfibrozil Alleviates Cognitive Impairment by Inhibiting Ferroptosis of Astrocytes via Restoring the Iron Metabolism and Promoting Antioxidant Capacity in Type 2 Diabetes. Mol. Neurobiol..

[214] Xu S.X., Xie X.H., Yao L., Wang W., Zhang H., Chen M.M., Sun S., Nie Z.W., Nagy C., Liu Z. (2023). Human in vivo evidence of reduced astrocyte activation and neuroinflammation in patients with treatment-resistant depression following electroconvulsive therapy. Psychiatry Clin. Neurosci..

[215] Park M.W., Cha H.W., Kim J., Kim J.H., Yang H., Yoon S., Boonpraman N., Yi S.S., Yoo I.D., Moon J.S. (2021). NOX4 promotes ferroptosis of astrocytes by oxidative stress-induced lipid peroxidation via the impairment of mitochondrial metabolism in Alzheimer’s diseases. Redox Biol..

[216] Mekhaeil M., Conroy M.J., Dev K.K. (2023). Elucidating the Therapeutic Utility of Olaparib in Sulfatide-Induced Human Astrocyte Toxicity and Neuroinflammation. J. Neuroimmune Pharmacol..

[217] Nakano-Kobayashi A., Canela A., Yoshihara T., Hagiwara M. (2023). Astrocyte-targeting therapy rescues cognitive impairment caused by neuroinflammation via the Nrf2 pathway. Proc. Natl. Acad. Sci. USA.

[218] Liu C., Zhao X.M., Wang Q., Du T.T., Zhang M.X., Wang H.Z., Li R.P., Liang K., Gao Y., Zhou S.Y. (2023). Astrocyte-derived SerpinA3N promotes neuroinflammation and epileptic seizures by activating the NF-kappaB signaling pathway in mice with temporal lobe epilepsy. J. Neuroinflamm..

[219] Yin S., Ma X.Y., Sun Y.F., Yin Y.Q., Long Y., Zhao C.L., Ma J.W., Li S., Hu Y., Li M.T. (2023). RGS5 augments astrocyte activation and facilitates neuroinflammation via TNF signaling. J. Neuroinflamm..

[220] Davaanyam D., Lee H., Seol S.I., Oh S.A., Kim S.W., Lee J.K. (2023). HMGB1 induces hepcidin upregulation in astrocytes and causes an acute iron surge and subsequent ferroptosis in the postischemic brain. Exp. Mol. Med..

[221] Ishii T., Warabi E., Mann G.E. (2019). Circadian control of BDNF-mediated Nrf2 activation in astrocytes protects dopaminergic neurons from ferroptosis. Free Radic. Biol. Med..

[222] Chen J., Qu X., Li Z., Zhang D., Hou L. (2019). Peak Neutrophil-to-Lymphocyte Ratio Correlates with Clinical Outcomes in Patients with Severe Traumatic Brain Injury. Neurocrit. Care.

[223] Li F., Weng G., Zhou H., Zhang W., Deng B., Luo Y., Tao X., Deng M., Guo H., Zhu S. (2024). The neutrophil-to-lymphocyte ratio, lymphocyte-to-monocyte ratio, and neutrophil-to-high-density-lipoprotein ratio are correlated with the severity of Parkinson’s disease. Front. Neurol..

[224] Xu C., Cai L., Yi T., Yi X., Hu Y. (2023). Neutrophil-to-lymphocyte ratio is associated with stroke progression and functional outcome in patients with ischemic stroke. Brain Behav..

[225] Nikaido Y., Midorikawa Y., Furukawa T., Shimoyama S., Takekawa D., Kitayama M., Ueno S., Kushikata T., Hirota K. (2022). The role of neutrophil gelatinase-associated lipocalin and iron homeostasis in object recognition impairment in aged sepsis-survivor rats. Sci. Rep..

[226] Liu Y.W., Li S., Dai S.S. (2018). Neutrophils in traumatic brain injury (TBI): Friend or foe?. J. Neuroinflamm..

[227] Wang K., Wang M., Liao X., Gao S., Hua J., Wu X., Guo Q., Xu W., Sun J., He Y. (2022). Locally organised and activated Fth1(hi) neutrophils aggravate inflammation of acute lung injury in an IL-10-dependent manner. Nat. Commun..

[228] Semple B.D., Trivedi A., Gimlin K., Noble-Haeusslein L.J. (2015). Neutrophil elastase mediates acute pathogenesis and is a determinant of long-term behavioral recovery after traumatic injury to the immature brain. Neurobiol. Dis..

[229] Garcia-Culebras A., Duran-Laforet V., Pena-Martinez C., Ballesteros I., Pradillo J.M., Diaz-Guzman J., Lizasoain I., Moro M.A. (2018). Myeloid cells as therapeutic targets in neuroinflammation after stroke: Specific roles of neutrophils and neutrophil-platelet interactions. J. Cereb. Blood Flow. Metab..

[230] Zhou J., Guo P., Hao X., Sun X., Feng H., Chen Z. (2023). Neutrophil Extracellular Traps (NETs): A New Therapeutic Target for Neuroinflammation and Microthrombosis After Subarachnoid Hemorrhage?. Transl. Stroke Res..

[231] Wang D., Yin K., Zhang Y., Lu H., Hou L., Zhao H., Xing M. (2023). Fluoride induces neutrophil extracellular traps and aggravates brain inflammation by disrupting neutrophil calcium homeostasis and causing ferroptosis. Environ. Pollut..

[232] Yao L., Sheng X., Dong X., Zhou W., Li Y., Ma X., Song Y., Dai H., Du Y. (2023). Neutrophil extracellular traps mediate TLR9/Merlin axis to resist ferroptosis and promote triple negative breast cancer progression. Apoptosis.

[233] Zhang H., Liu J., Zhou Y., Qu M., Wang Y., Guo K., Shen R., Sun Z., Cata J.P., Yang S. (2022). Neutrophil extracellular traps mediate m(6)A modification and regulates sepsis-associated acute lung injury by activating ferroptosis in alveolar epithelial cells. Int. J. Biol. Sci..

[234] Lv T., Xiong X., Yan W., Liu M., Xu H., He Q. (2023). Mitochondrial general control of amino acid synthesis 5 like 1 promotes nonalcoholic steatohepatitis development through ferroptosis-induced formation of neutrophil extracellular traps. Clin. Transl. Med..

[235] Galicia G., Boulianne B., Pikor N., Martin A., Gommerman J.L. (2013). Secondary B cell receptor diversification is necessary for T cell mediated neuro-inflammation during experimental autoimmune encephalomyelitis. PLoS ONE.

[236] Hartlehnert M., Borsch A.L., Li X., Burmeister M., Gerwien H., Schafflick D., Heming M., Lu I.N., Narayanan V., Strecker J.K. (2021). Bcl6 controls meningeal Th17-B cell interaction in murine neuroinflammation. Proc. Natl. Acad. Sci. USA.

[237] Mundt S., Mrdjen D., Utz S.G., Greter M., Schreiner B., Becher B. (2019). Conventional DCs sample and present myelin antigens in the healthy CNS and allow parenchymal T cell entry to initiate neuroinflammation. Sci. Immunol..

[238] Rayasam A., Kijak J.A., Dallmann M., Hsu M., Zindl N., Lindstedt A., Steinmetz L., Harding J.S., Harris M.G., Karman J. (2018). Regional Distribution of CNS Antigens Differentially Determines T-Cell Mediated Neuroinflammation in a CX3CR1-Dependent Manner. J. Neurosci..

[239] Harrer C., Otto F., Pilz G., Haschke-Becher E., Trinka E., Hitzl W., Wipfler P., Harrer A. (2021). The CXCL13/CXCR5-chemokine axis in neuroinflammation: Evidence of CXCR5+CD4 T cell recruitment to CSF. Fluids Barriers CNS.

[240] Williams J.L., Manivasagam S., Smith B.C., Sim J., Vollmer L.L., Daniels B.P., Russell J.H., Klein R.S. (2020). Astrocyte-T cell crosstalk regulates region-specific neuroinflammation. Glia.

[241] Luoqian J., Yang W., Ding X., Tuo Q.Z., Xiang Z., Zheng Z., Guo Y.J., Li L., Guan P., Ayton S. (2022). Ferroptosis promotes T-cell activation-induced neurodegeneration in multiple sclerosis. Cell Mol. Immunol..

[242] Gross C.C., Meyer C., Bhatia U., Yshii L., Kleffner I., Bauer J., Troscher A.R., Schulte-Mecklenbeck A., Herich S., Schneider-Hohendorf T. (2019). CD8(+) T cell-mediated endotheliopathy is a targetable mechanism of neuro-inflammation in Susac syndrome. Nat. Commun..

[243] Liang J., Shen Y., Wang Y., Huang Y., Wang J., Zhu Q., Tong G., Yu K., Cao W., Wang Q. (2022). Ferroptosis participates in neuron damage in experimental cerebral malaria and is partially induced by activated CD8(+) T cells. Mol. Brain.

[244] Laurent C., Dorothee G., Hunot S., Martin E., Monnet Y., Duchamp M., Dong Y., Legeron F.P., Leboucher A., Burnouf S. (2017). Hippocampal T cell infiltration promotes neuroinflammation and cognitive decline in a mouse model of tauopathy. Brain.

[245] Yshii L., Pasciuto E., Bielefeld P., Mascali L., Lemaitre P., Marino M., Dooley J., Kouser L., Verschoren S., Lagou V. (2022). Astrocyte-targeted gene delivery of interleukin 2 specifically increases brain-resident regulatory T cell numbers and protects against pathological neuroinflammation. Nat. Immunol..

